# Porous Silicon Nanoparticle Hydrogel Formulation for Topical Delivery of Flightless I Neutralizing Antibody and Improved Diabetic Wound Healing

**DOI:** 10.1002/smll.202505359

**Published:** 2025-08-27

**Authors:** Christopher T. Turner, Parham Sahandi Zangabad, Kiralee Janusaitis, Nikki Black, Robert Fitridge, Allison J. Cowin, Nicolas H. Voelcker

**Affiliations:** ^1^ Drug Delivery, Disposition and Dynamics, Monash Institute of Pharmaceutics Science Monash University Parkville Campus Parkville VIC 3052 Australia; ^2^ Melbourne Centre for Nanofabrication Victorian Node of the Australian National Fabrication Facility Clayton VIC 3168 Australia; ^3^ Regenerative Medicine Future Industries Institute University of South Australia Adelaide SA 5095 Australia; ^4^ Faculty of Health and Medical Sciences University of Adelaide Adelaide SA 5005 Australia; ^5^ Department of Materials Science and Engineering Monash University Clayton VIC 3800 Australia

**Keywords:** chronic diabetic wounds, porous silicon nanoparticles, slow release, therapeutic antibody, topical drug delivery

## Abstract

Diabetes‐related foot ulcers (DRFUs), affecting one‐third of individuals with diabetes, are a leading cause of hospitalization. Current treatment options to expedite healing are limited, with the effectiveness of potential therapeutics reduced by the hostile environment inherent to most diabetes‐related wounds. Topical delivery vehicles that protect drugs from the protease‐enriched wound environment, whilst displaying a slow‐release profile, are needed to facilitate wound closure. Biodegradable porous silicon nanoparticles (pSi NPs) in a thermo‐sensitive hydrogel formulation are assessed for the delivery of the wound healing therapeutic Flightless I neutralizing antibody (FnAb). FnAb is loaded into pSi NPs at clinically relevant doses and achieves functional antibody release over 14 days. In vitro, pSi NPs effectively protected FnAb from proteolytic degradation by matrix metalloproteinases that are elevated in diabetes‐related wounds. FnAb‐loaded pSi NPs are formulated into a thermo‐responsive hydrogel and delivered topically to type 1 and type 2 diabetic mouse wounds. The hydrogel facilitated FnAb release from pSi NPs until absorbed into the wound bed. A single topical application led to reduced Flightless I, improved wound closure, decreased inflammation, and improved tissue remodeling compared to the delivery of FnAb alone. Together, pSi NP hydrogel is an effective vehicle to deliver therapeutic FnAb to diabetes‐related wounds.

## Graphical abstract

A biodegradable porous silicon nanoparticle (pSi NP) hydrogel formulation delivers a Flightless I neutralizing antibody (FnAb) topically to diabetic wounds. The formulation protects FnAb from proteolytic degradation and enables sustained 14‐day release, aligned with routine dressing changes. This platform promotes wound closure, reduces inflammation, and offers direct topical applicability and strong translational potential for antibody‐based treatment of chronic diabetic wounds.

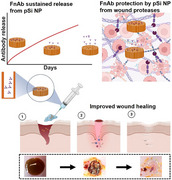

## 1 Introduction

The global burden of diabetes is substantial, with over 537 million people affected worldwide, 90% of whom are attributed to type 2 diabetes. The prevalence is increasing and projected to reach over 780 million cases by 2045, mainly due to an aging population, increased obesity, and sedentary lifestyle.^[^ ^1^ ^]^ An important complication is the development of diabetes‐related foot ulcers (DRFUs), with 19% to 34% of people expected to develop one in their lifetime.^[^ ^2^, ^3^ ^]^ Twenty percent of people with DRFUs will need an amputation of their lower extremity.^[^ ^3^, ^4^ ^]^ A major amputation due to diabetes‐related foot complications has a mortality rate of over 50% within 5 years.^[^ ^4^, ^5^ ^]^ This growing clinical challenge is reflected in the expanding market for treatments, projected to reach US$7 billion in 2019 to US$11 billion by 2027.^[^ ^6^ ^]^

Current pharmaceutical treatments are in high demand; however, options remain limited. Topical therapeutics aim to improve DRFU healing by facilitating the transition from a chronic non‐healing phenotype where wounds become “stalled” in a pro‐inflammatory state to successfully closed wounds.^[^ ^7^ ^]^ Multiple targets within the inflammatory cascade (i.e., metalloproteinases; MMP) and repair mechanisms (i.e., platelet‐derived growth factor; PDGF) have been identified as potential drug targets. Pan‐specific MMP inhibitors have been unsuccessful, and only Regranex, a PDGF inhibitor, has gained FDA approval for the treatment of diabetic wounds.^[^ ^8^ ^]^ However, this drug has demonstrated side effects associated with long‐term use and has had limited market adoption.

A major limitation in the development of topical therapeutics for DRFUs is the chronic wound environment,^[^ ^9^, ^10^ ^]^ which is characterized by altered pH and up to 100‐fold increase in protease levels.^[^ ^11^ ^]^ One important protease group, MMPs, has ≥30‐fold higher activity in chronic than acute wounds,^[^ ^12^ ^]^ and these can directly damage therapeutic antibodies (i.e., MMP2, MMP3, and MMP9).^[^ ^13^, ^14^ ^]^ The hostile environment is further complicated by the presence of bacterial infection, which supplies an additional protease load.^[^ ^15^, ^16^ ^]^ Although therapeutics may still be effective, proteases will likely reduce the therapeutic agents half‐life, thereby limiting their efficacy. Hence, there is a critical need to develop innovative formulations capable of effectively delivering and retaining active therapeutics within the DRFU environment.

Porous silicon (pSi) is a promising carrier for drug delivery applications due to its high porosity (up to 80%) and surface area (up to 800 m^2^ g^−1^), as well as in vivo biocompatibility and biodegradability.^[^ ^17^, ^18^ ^]^ Particle size, pore diameter, and surface chemistry of pSi are highly tunable, resulting in high drug loading capacity, controlled particle degradation, and drug release kinetics.^[^ ^19^ ^]^ Our research group has extensively studied pSi NP tailored for the delivery of various therapeutics including siRNA,^[^ ^20^, ^21^ ^]^ antibodies,^[^ ^22^, ^23^ ^]^ small molecule,^[^ ^21^, ^24^ ^]^ and peptides.^[^ ^25^ ^]^ We have reported the use of various surface modifications to control pSi degradation rates and surface charge, directly influencing drug loading and release profiles.^[^ ^22^, ^23^, ^25^, ^26^, ^27^, ^28^ ^]^ Specifically, we have established pSi surface modifications, such as controlled oxidation, suitable for effective delivery of bioactive therapeutics.^[^ ^22^, ^23^, ^28^, ^29^ ^]^ pSi carriers have also been shown to deliver therapeutic agents while protecting their structural integrity and therapeutic activity in hostile biological environments.^[^ ^22^, ^30^ ^]^ The in vivo and in vitro biocompatibility and biodegradability of pSi have been demonstrated by us^[^ ^18^, ^21^, ^31^, ^32^, ^33^ ^]^ and others,^[^ ^34^, ^35^, ^36^, ^37^, ^38^, ^39^, ^40^, ^41^ ^]^ with biodistribution studies displaying a lack of toxicity or inflammation. Silicon, a vital element in the body's metabolic processes, is typically present in the form of orthosilicic acid, which is readily excreted by the kidneys.^[^ ^42^ ^]^ Orthosilicic acid is generated by the oxidative hydrolysis of pSi in aqueous media.^[^ ^43^, ^44^ ^]^ Biodegradation of silicon has been found to begin from the external surfaces of pSi particle carriers, along with dissolving the pore walls inside the pores.^[^ ^41^ ^]^ The release of therapeutic payloads occurs as the pSi gradually degrades from external and inner pore surfaces, through an increase in pore size and decrease in pSi volume and density over time,^[^ ^41^ ^]^ rather than solely through diffusion out of the pores,^[^ ^45^ ^]^ thereby linking the rate of degradation to drug release. In our previous work, we demonstrated the effective loading and release of infliximab, an IgG monoclonal antibody that inhibits tumor necrosis factor‐α, from pSi carriers.^[^ ^23^ ^]^ Here, we provided proof‐of‐concept for efficient loading and the controlled release of functional antibody, suggesting pSi may be adapted to wound treatment applications.

Flightless I is an actin remodeling protein and a negative regulator of wound healing.^[^ ^46^, ^47^, ^48^, ^49^, ^50^ ^]^ Identified as a therapeutic target for diabetes‐related wound care, anti‐Flightless I neutralizing antibodies (FnAb) improved wound healing in healthy mice^[^ ^51^ ^]^ and pigs.^[^ ^48^ ^]^ In type 1 diabetic mice, intradermal injection of FnAb improved wound healing and the rate of re‐epithelialization.^[^ ^52^, ^53^ ^]^ The genetic reduction of Flii in type 1 diabetic mice corresponded to reduced inflammation, including decreased detection of both neutrophils and macrophages.^[^ ^52^ ^]^ Based on these wound healing properties, FnAb has considerable potential for development as a treatment for DRFUs, however, it is susceptible to proteolytic degradation, and thus delivery in pSi NPs is of great interest.

**Figure 1:**
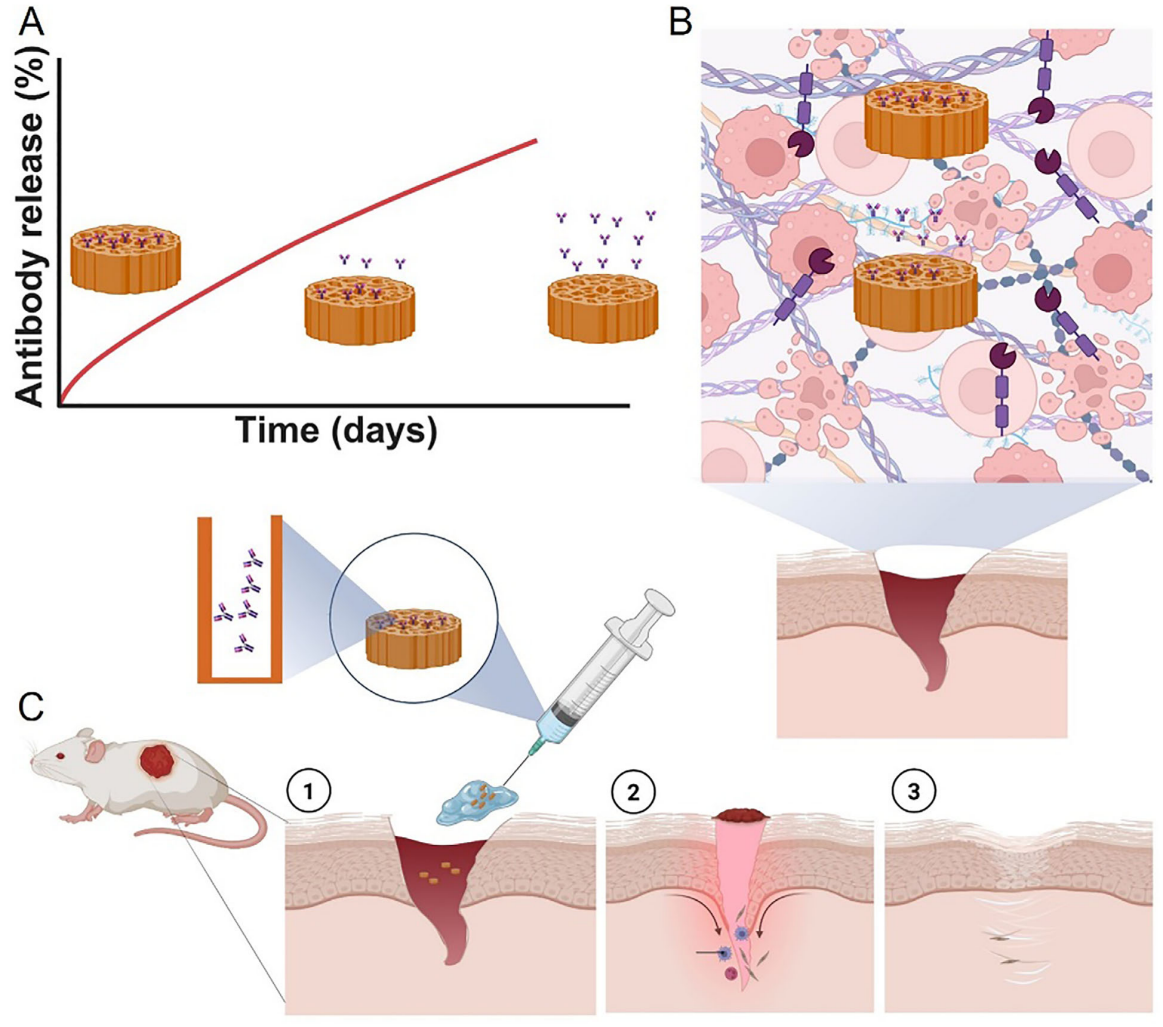
FnAb pSi NP hydrogel to reduce Flightless I, improve wound closure, decrease inflammation, and enhance tissue remodeling in diabetic wounds. A) Sustained release of FnAb from pSi NP carrier. B) pSi NP protection of FnAb from proteases enriched in the diabetic wound environment. C) Topical FnAb delivery to type 2 diabetic mouse wounds using pSi NP carriers formulated into a thermo‐sensitive hydrogel, subsequently improving wound healing. Reproduced with permission from BioRender.

Previously, we loaded and released FnAb from pSi NPs, overcoming the challenge of retaining functionality when bound to pSi or upon release.^[^ ^22^ ^]^ Further investigation identified the ability of pSi to protect loaded FnAb from pepsin, a stomach protease known to cleave IgG in acid‐rich environments. Finally, a proof‐of‐concept assessment of intradermally injected FnAb‐loaded pSi NPs (FnAb pSi NPs) in wounds of type 1 diabetic mice was performed with more rapid wound closure observed.^[^ ^22^ ^]^ Intradermal injection of therapeutics to wounds is likely to have limited clinical uptake due to the increasing risk of irritation and microinjuries,^[^ ^54^ ^]^ the inability to sustain localized therapeutic concentrations (i.e., elevated systemic concentration of drugs, leading to side effects and reduced treatment effectiveness),^[^ ^55^ ^]^ and the availability of advanced alternatives (such as topical hydrogels) with superior efficacies, reduced patient discomfort, and better suitability for clinical use.^[^ ^56^, ^57^ ^]^ Therefore, in this current study, we have developed a clinically relevant FnAb pSi NP thermo‐sensitive gel formulation and tested its ability to topically deliver functional antibody to improve healing in both type 1, but most importantly, type 2 diabetes, the population cohort accounting for a significant proportion of all DRFUs. We assessed the capacity for sustained release of functional antibody (**Figure** **1**A) and protection of FnAb from proteases, including MMPs (Figure 1B) that are specifically elevated in diabetic wounds. We optimized the thermo‐sensitive hydrogel formulation to topically deliver FnAb pSi NP to mouse models of type 1 and type 2 diabetic wounding (Figure 1C). Finally, we investigated the capacity of the FnAb pSi NP hydrogel to reduce its target Flightless I, improve wound closure, reduce inflammation, and enhance tissue remodeling in these diabetic models.

## 2 Results and Discussion

### 2.1 Fabrication and Characterization of pSi NPs

**Figure 2:**
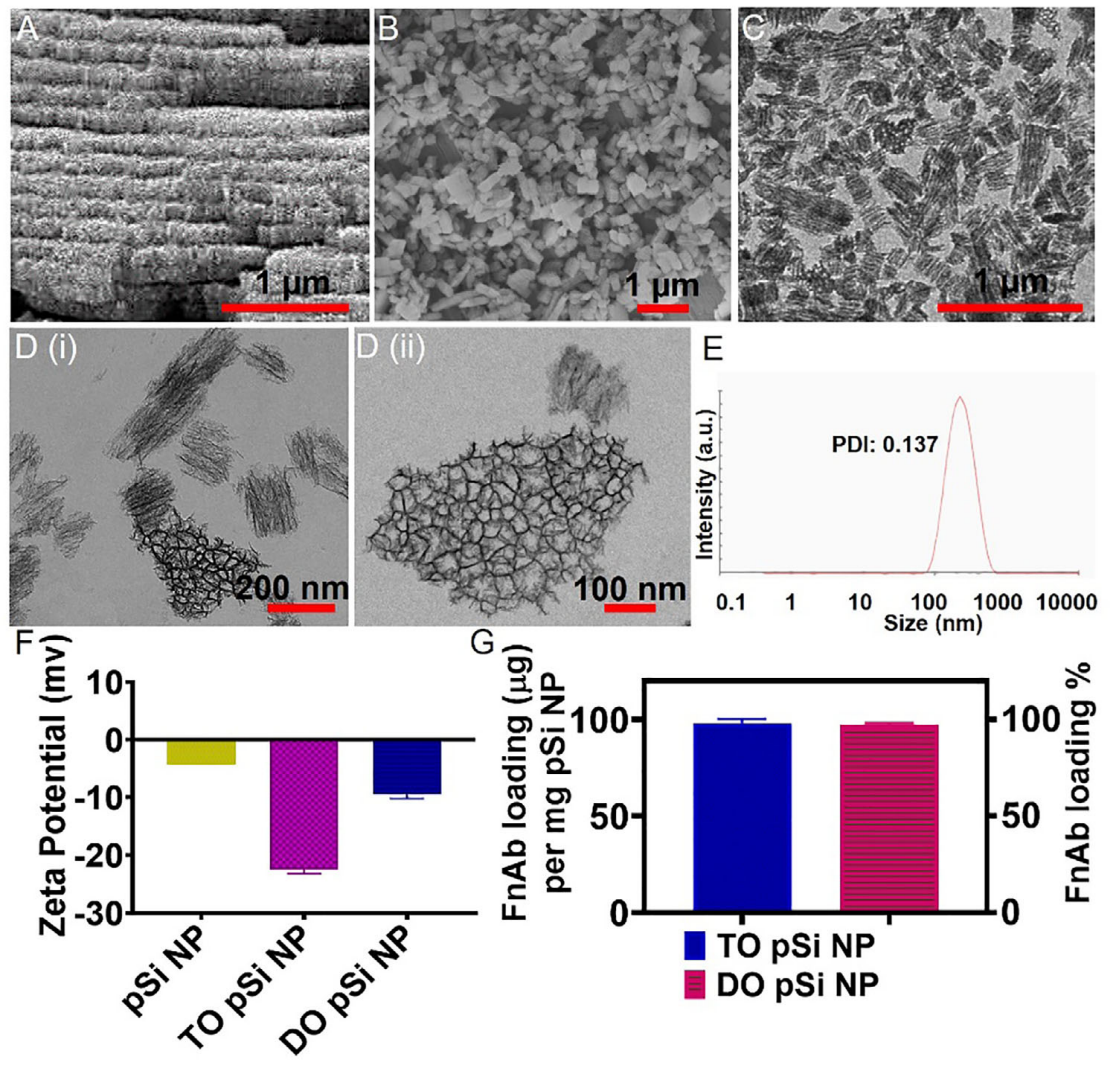
Generation of pSi NP carriers with selected pore size and particle size of interest. A) FEG‐SEM image of pSi multilayer films produced by electrochemical etching of Si wafer using etching condition of 50 mA cm^−2^ for 4.4 s in 3:1 HF: ethanol. FEG‐SEM B), and TEM C) images of size‐selected pSi NPs with an average size of 174 nm. D) i) and ii) High‐resolution TEM images at higher magnification showing pSi NPs with the pore size of 35 nm. E) pSi NP size measured by dynamic light scattering (DLS) with average particle size: 206 nm, PDI: 0.137. F) pSi NP Zeta potential measurements. G) Loading (µg) of FnAb per mg of TO‐pSi NPs or DO‐pSi NPs (initial loading condition: 1 mg FnAb in 10 mg pSi, 4 °C). Data in (F,G) presented as mean ± SD (*n* = 3 per group).

pSi NPs were fabricated using a two‐step electrochemical etching process (50 mA cm^−^^2^ for 4.3 s, followed by 188 mA cm^−^^2^ for 0.2 s) (**Figure** **2**A), generating multilayer films with layer thickness of ≈190 nm via a wet bench setup (Figure S1, Supporting Information). Field emission gun scanning electron microscopy (FEG‐SEM) and transmission electron microscopy (TEM) confirmed the formation of pSi NPs with an average size of 174 nm (Figure 2B,C) and pore size of 35 nm according to high‐resolution TEM images (Figure 2D). Dynamic light scattering (DLS) analysis yielded a hydrodynamic diameter of 206 nm (PDI 0.137) (Figure 2E), slightly larger than the TEM value, because DLS measures the hydrodynamic diameter of NPs, reflecting the inclusion of the hydration layer around the NPs. Next, pSi NPs were modified with thermal oxidation (TO) and dimethyl sulfoxide (DMSO) oxidation (DO). Zeta potential measurements revealed a more negative surface charge for TO pSi NPs (−25 mV) compared to DO pSi NPs (−9 mV) and unmodified pSi NPs (−4 mV) (Figure 2F).

### 2.2 Loading of FnAb into pSi NPs at Clinically Relevant Doses

We first assessed the loading efficiency of FnAb into pSi NPs at neutral pH 7.4 in PBS as this was most likely to help retain antibody functionality. Oxidized pSi NPs have been established as suitable carriers for IgG antibody loading and release^[^ ^58^ ^]^ with the oxidation method influencing NP stability and drug adsorption into NPs.^[^ ^23^ ^]^ As FnAb is an IgG antibody,^[^ ^59^, ^60^ ^]^ both TO and DO methods were investigated for FnAb loading. TO is a more stable modification compared to milder DO, with DO modification being prone to rapid dissolution in aqueous media.^[^ ^61^ ^]^ Both formulations exhibited near‐complete FnAb loading efficiency, with 98.0 ± 0.2 and 97.2 ± 0.8 µg mg^−1^ (mean ± SD, *n* = 3) for TO and DO modifications, respectively, at clinically relevant doses (Figure 2G), achieved by incubating 1 mg FnAb (1 mg mL^−1^) with 10 mg pSi NPs overnight at 4 °C and quantified by means of UV–vis spectroscopy.

We subsequently evaluated the effect of varying pH conditions on FnAb loading efficiency, including an acidic pH of ≈6 (in acetate buffer), a neutral pH of 7.4 (in PBS), and an alkaline pH of ≈8 (in Tris buffer). The FnAb was incubated with TO pSi NPs in each buffer condition. FnAb loading efficiencies were found to be 99.3 ± 0.15, 99.0 ± 0.2, and 96.4 ± 0.2 µg mg^−1^ at pH 7.4, pH 6, and pH 8, respectively. Despite statistical significances in loading between groups, the absolute difference in loading efficiencies was relatively small, suggesting that the practical impact of varying the pH from 6 to 8 on FnAb loading within the tested range remains minimal. This finding is consistent with our previous studies reporting that pH variations have minimal impact on antibody loading into oxidized pSi NPs.^[^ ^23^ ^]^ Furthermore, deviations from neutral pH pose a risk of structural compromise and loss of biological function, as antibodies are known to denature or aggregate under acidic or highly basic conditions.^[^ ^62^, ^63^ ^]^

Taken together, these results suggest that the efficient loading of FnAb into pSi NPs cannot be solely attributed to electrostatic interactions between the negatively charged pSi NP surface (at neutral pH)^[^ ^61^ ^]^ and FnAb with a near neutral pH isoelectric point (pI).^[^ ^59^ ^]^ In addition to electrostatic forces,^[^ ^64^ ^]^ other mechanisms likely contribute to the high loading efficiency of FnAb into pSi NPs including physical entrapment (steric loading),^[^ ^61^, ^65^, ^66^ ^]^ hydrophobic interactions (particularly in DO pSi NPs, due to residual Si─H bonds), localized charge patches on the protein surface,^[^ ^64^ ^]^ van der Waals and capillary forces, and hydrogen bonding between the antibody and the oxidized pSi surface.^[^ ^67^, ^68^ ^]^ The combined effect of these mechanisms likely maintains consistently high loading across the different pH conditions tested. Of note, the obtained average pore size of ≈35 nm in this study falls within the optimal range (≈20–40 nm) previously reported for efficient antibody loading into pSi NPs.^[^ ^22^, ^23^, ^29^, ^69^ ^]^ Pores smaller than this range may restrict antibody entry, while significantly larger pores would not be expected to further enhance loading efficiency.

### 2.3 pSi NPs Degrade in Simulated Wound Fluid

**Figure 3:**
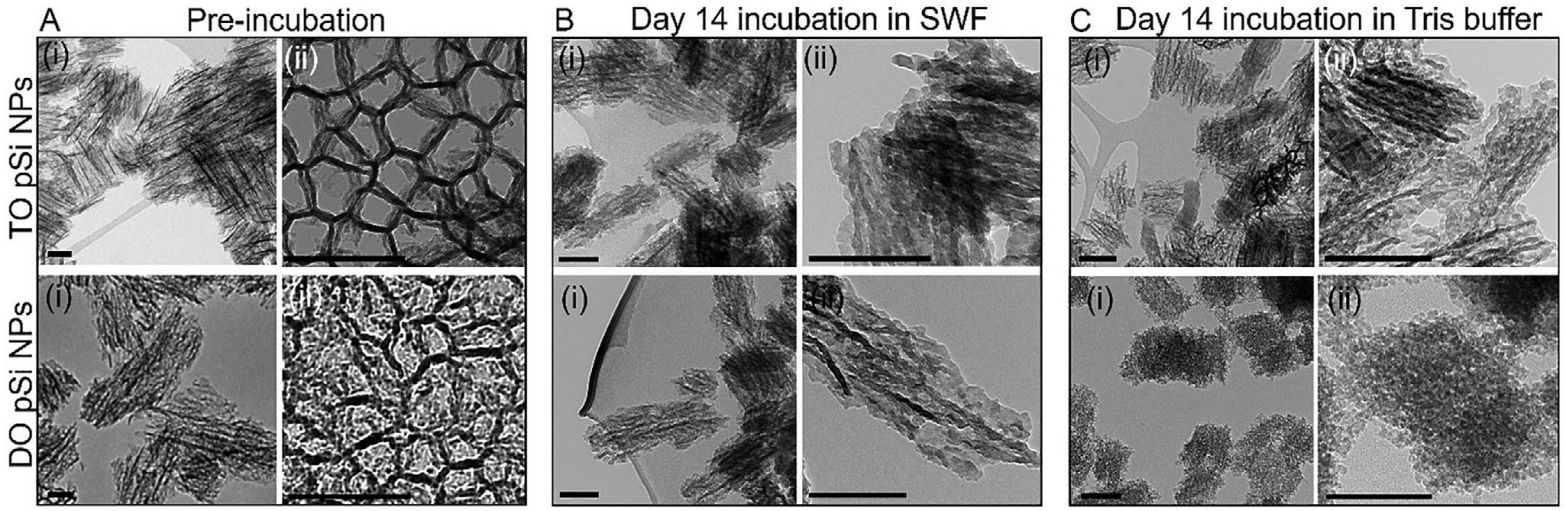
pSi NPs degrade under simulated wound conditions. TEM images of TO pSi NPs and DO pSi NPs before (A) and after incubation in SWF (1:1 human serum: human plasma) (B) or Tris buffer (0.1% Tween 20, 5% (w/v) BSA) (C) at 34 °C for 14 days. Images were taken to assess the structural changes and degradation rate of the NPs. As pSi NP degradation is required for payload release, this test aims to evaluate the potential of pSi NPs as a sustained release system for FnAb in a wound environment. Size bars = 100 nm.

The degradation of TO and DO pSi NPs, required for FnAb release, was compared when incubated in simulated wound fluid (SWF, 1:1 human serum: human plasma) or Tris buffer containing 0.1% Tween 20, 5% (w/v) bovine serum albumin (BSA), at 34 °C (Figure S2, Supporting Information). TEM images revealed the time‐dependent degradation of pSi NPs incubated in the SWF and Tris buffer, showing structural changes over 14 days (**Figure** **3**). At day 14, changes in the morphology of DO and TO pSi NPs were observed, including the loss of defined edges (blurred or irregular pore boundaries) in the NP porous structure and the appearance of more irregular and fragmented NP shapes.^[^ ^70^, ^71^ ^]^ These changes, indicative of facilitated pSi NP degradation, were more pronounced for DO pSi NPs than TO pSi NPs and were more significant in Tris buffer than in SWF. This suggests that TO pSi NPs exhibit a more stable structure than DO pSi NPs. DO, as a mild oxidation, forms a thin, hydrophilic Si─O─Si layer while preserving some residual Si─H bonds.^[^ ^72^ ^]^ TO leads to partial back‐bond oxidation (more stable layers of Si─O─Si) with Si─OH species on the surface.^[^ ^72^ ^]^ Si─Si and Si─H bonds present in pSi are inherently less stable (i.e., accelerated hydrolysis) than their oxidized counterparts, Si─O─Si and Si─OH.^[^ ^73^ ^]^ SWF is a protein‐rich buffer providing a neutral pH (≈7.4). Proteins in this buffer can be adsorbed onto pSi surfaces, protecting pSi and slowing down pSi degradation. The protective effect from protein adsorption is absent for Tris buffer, which also has a higher pH of 8.3, explaining for observed faster degradation.^[^ ^74^ ^]^

### 2.4 pSi NPs Release Functional FnAb at a Clinically Relevant Rate

**Figure 4:**
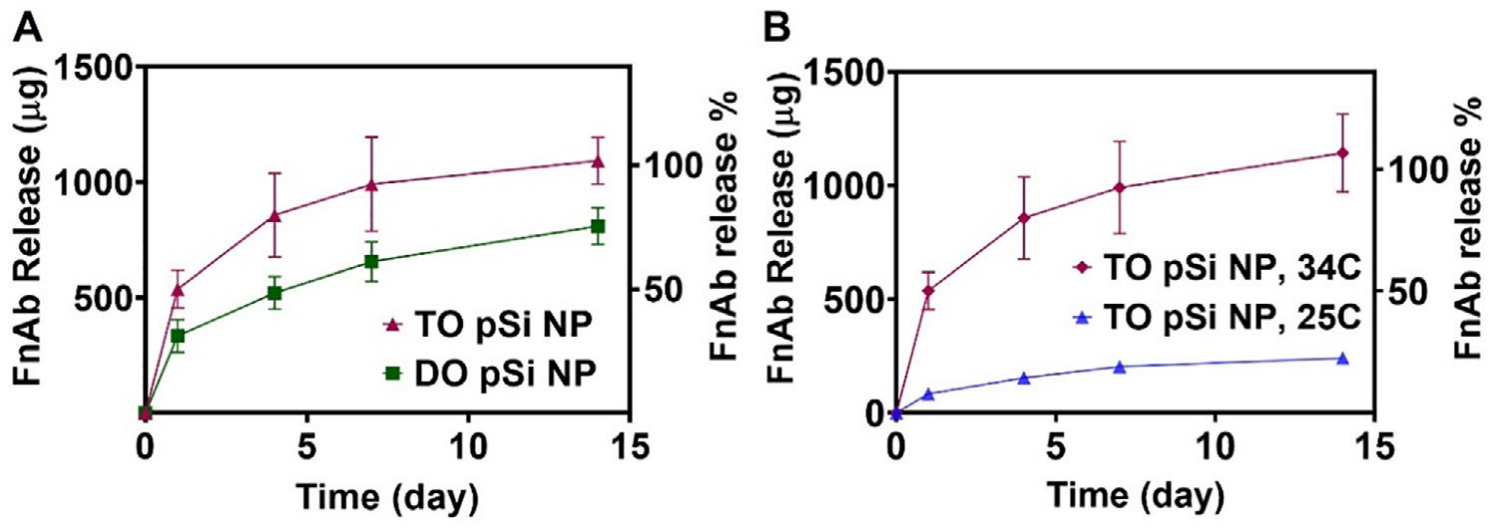
Efficient release of functional FnAb from TO and DO pSi NPs. A) Cumulative FnAb release from TO pSi NPs and DO pSi NPs incubated in SWF for 14 days at 34 °C. B) Cumulative FnAb release from TO pSi NPs incubated in SWF for 14 days at 25 and 34 °C. FnAb present in the release samples was quantified by ELISA, with only functional antibody detected. All data is presented as mean ± STD (*n* = 3).

DRFUs provide a complex protein‐enriched wound fluid environment, thus FnAb release was assessed in SWF to accurately reflect release kinetics in a wound environment. Conventional spectroscopy methods (e.g., UV‐vis) cannot selectively measure FnAb release in this medium, thus FnAb release was measured using an FnAb‐specific indirect ELISA (Figure S3A, Supporting Information). This in‐house assay was created to allow detection of structurally intact antibody (able to bind its target, recombinant human Flightless I protein), thereby providing important information regarding FnAb's functionality.^[^ ^22^ ^]^ A calibration curve in the ELISA allowed quantification of FnAb (Figure S3B,C, Supporting Information). In SWF, ≈50% FnAb was released from TO pSi NPs within the first 24 h, then there was a gradual reduction in release rate up to 14 days (**Figure** **4**A). One hundred percent was released by day 14 with all of it maintaining functionality throughout loading and release. This observation aligns with our previous studies, which demonstrated IgG antibodies such as FnAb and infliximab to exhibit sustained release from TO pSi NPs and TO pSi microparticles.^[^ ^22^, ^23^ ^]^ In contrast, 33% of functional FnAb was released from DO pSi NPs over the first 24 h followed by only 80% of functional FnAb released by day 14, suggesting incomplete release or partial damage to antibody functionality.

The enhanced release of functional FnAb from TO pSi NPs compared to DO pSi NPs is likely attributable to the facilitated diffusion of functional FnAb through the pores of TO pSi NPs. This aligns with prior reports demonstrating that oxidation methods affect pSi degradation, and drug molecule loading and release properties.^[^ ^23^ ^]^ Despite TO pSi NPs exhibiting greater structural stability than DO pSi NPs (Figure 3), their superior functional FnAb release performance can be correlated to the surface chemistry differences. Mildly oxidized DO pSi, with residual Si─H bonds, is more prone to hydrolysis than more stable TO pSi with surface Si─OH bonds.^[^ ^73^ ^]^ Moreover, the lower stability of pSi nanostructures, such as DO pSi NPs, along with reactive residual Si─H species on the surface, create a reducing environment. This can cause redox degradation of biological payloads, hence, potentially inactivating and damaging their structure either before or during release from NPs.^[^ ^75^, ^76^ ^]^ In contrast, more stable oxidized pSi NPs, such as TO can attenuate redox reactions and the subsequent drug degradation, thus, preserving the drug's functionality.^[^ ^76^ ^]^

The effect of temperature on FnAb release from TO pSi NPs in SWF was investigated. When incubated at 25 °C compared to 34 °C, there was a fivefold reduction in FnAb release (Figure 4B). This temperature dependence is consistent with diffusion‐controlled release mechanisms, where increased molecular mobility at higher temperatures facilitates antibody diffusion from the pSi NP pores. This finding suggests that TO pSi NPs in SWF at 34 °C would provide optimal FnAb release kinetics under in vivo wound conditions (with a typical temperature of 30–33 °C). Together, due to the better performance of TO than DO modification, we moved forward with TO pSi NP (now referred to as pSi NP).

Performing release kinetics tests in SWF^[^ ^77^, ^78^ ^]^ highlights the importance of assessing drug release using in vitro conditions representative of its final clinical application (clinical wound environments).^[^ ^77^, ^79^, ^80^, ^81^ ^]^ Suitable release media conditions,^[^ ^77^, ^80^, ^81^, ^82^, ^83^, ^84^ ^]^ such as medium temperature, viscosity, and protein content, play a crucial role in obtaining more relevant antibody release kinetics and in maintaining the functionality of released antibodies.

To investigate the dominant release mechanism of FnAb from pSi NPs, the cumulative release profiles at 34 °C presented in Figure 4A (as this temperature is more clinically relevant) were fitted to several well‐established mathematical models, including Zero‐order, First‐order, Higuchi, and Korsmeyer‐Peppas.^[^ ^25^ ^]^ The fitting results are summarized in Table S1 (Supporting Information).

The Korsmeyer‐Peppas model provided the best fit for both TO pSi NPs and DO pSi NPs, with R^2^ values of 0.98 and 0.98, respectively. The release exponent (n) values obtained from this model were 0.25 and 0.34 for TO pSi NPs and DO pSi NPs, respectively. As n values below 0.43 are characteristic of Fickian diffusion, these results indicate that the release of FnAb from pSi NPs is primarily governed by diffusion‐controlled mechanisms. This conclusion was further supported by reasonable fits to the First‐order and Higuchi models, which describe release mechanisms driven by both drug concentration gradients and diffusion, although the Korsmeyer‐Peppas model demonstrated superior accuracy. The Zero‐order model provided a poor fit (R^2^ = 0.66 and 0.79 for TO pSi NPs and DO pSi NPs, respectively), suggesting that constant release or particle degradation mechanisms are less likely to dominate in the release. These findings are consistent with the porous structure of pSi NPs and the diffusional transport of drug molecules in porous carriers, along with the degradation behavior of pSi NP observed over 14 days (Figure 3). Taken together, the mathematical model fitting analysis confirms that the release of FnAb from pSi NPs is best described by Fickian diffusion (i.e., concentration gradient‐induced) within the tested timeframe.

### 2.5 pSi NPs Protect Loaded FnAb Against Proteolytic Degradation

**Figure 5:**
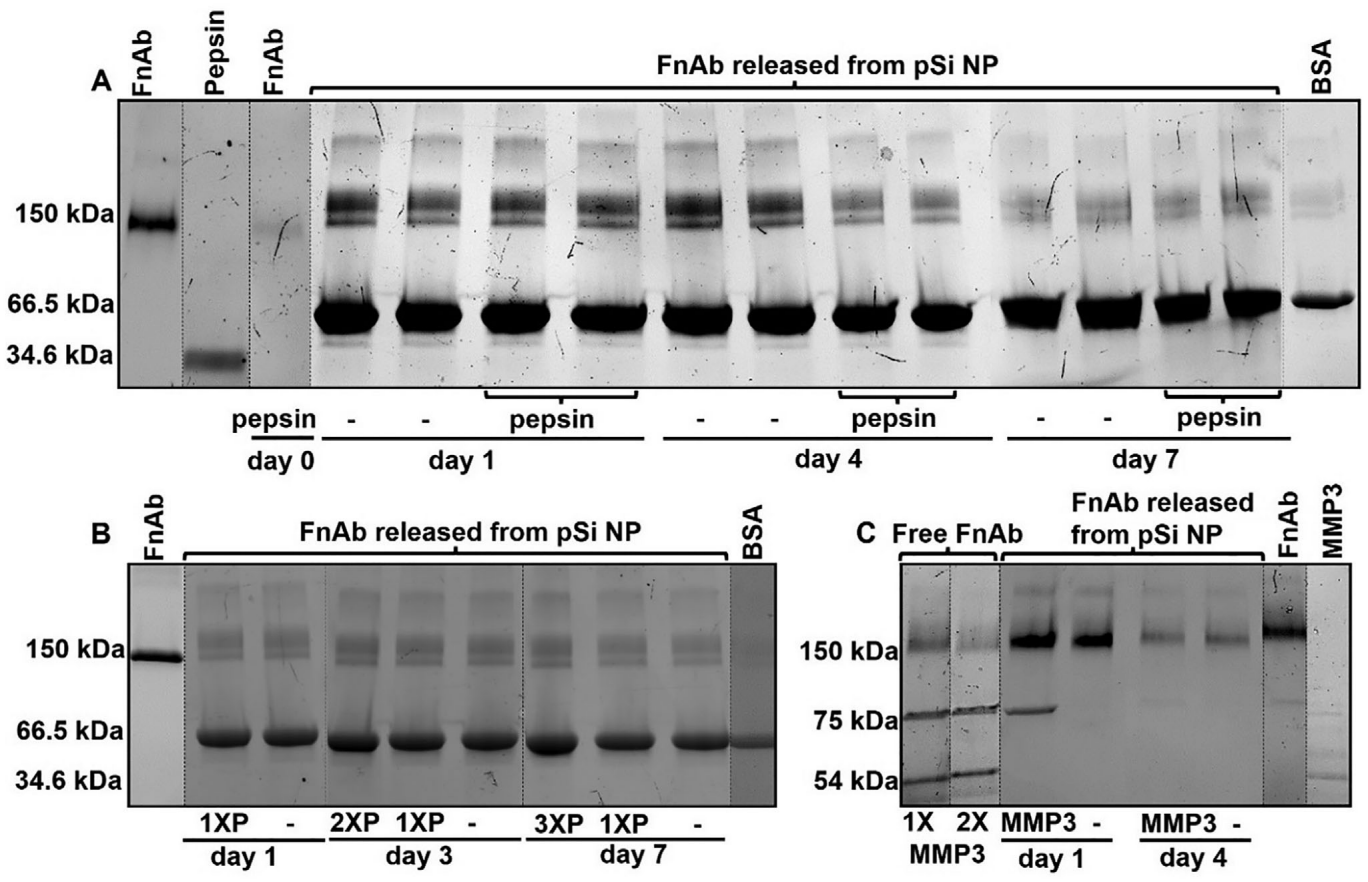
pSi NP protection of FnAb from proteolytic degradation. A series of experiments were conducted where FnAb pSi NPs were transiently exposed to proteases, washed, then a release experiment was performed. Supernatants were collected throughout and run on an SDS‐PAGE gel to assess the structural integrity of the antibody. A) FnAb pSi NPs exposed to pepsin, B) multiple rounds of pepsin, and C) MMP3. Non‐degraded FnAb is observed as a 150 kDa band. Proteolytic cleavage leads to an increase in lower molecular weight bands. The 66.5 kDa band represents BSA, which is included in the release buffer to stabilize FnAb (A,B). In Figure 5A–C, lanes from the same gel were rearranged for clarity (splicing indicated by vertical dashed lines); all samples within each experiment were run under identical conditions.

To assess the susceptibility of FnAb in pSi NP to proteolytic degradation, FnAb pSi NPs were transiently exposed to pepsin, a well‐documented protease known to cleave IgG antibodies.^[^ ^85^, ^86^ ^]^ Following pepsin exposure, pSi was pelleted, pepsin‐containing supernatant discarded, and the pellet was resuspended in release buffer (Tris buffer, 0.1% Tween 20, 5% (w/v) BSA) and incubated for 7 days. Supernatant aliquots were collected on 1, 4, and 7 days, and FnAb size was assessed on SDS‐PAGE gel (**Figure** **5**A). At each time point, FnAb released from pepsin‐treated pSi NPs exhibited a 150‐kDa band, indicating intact antibody structure, consistent with the non‐pepsin exposed control. In contrast, free FnAb (i.e., not loaded into pSi NPs) subjected to the same pepsin digestion conditions showed almost complete degradation, as evidenced by the faint 150‐kDa band, validating the efficacy of the digestion protocol. No pepsin band was detected on 1, 4, or 7 days, confirming effective pepsin removal prior to the release experiment.

In the next experiment, FnAb‐loaded pSi NPs were subjected to 1, 2, or 3 cycles of pepsin exposure (1XP, 2XP, 3XP, respectively) at day 0, day 1, and day 3, respectively (see methods section for more detail). As above, pSi NPs were washed in Tris buffer between each pepsin exposure and release experiment. Supernatant aliquots were collected on 1, 3, and 7 days for analysis (Figure 5B). SDS‐PAGE analysis revealed that FnAb released from the FnAb‐pSi NPs after one, two, or three pepsin exposure cycles displayed a distinct 150 kDa band, providing further support that loading within pSi NPs protected FnAb from pepsin degradation.

Pepsin is not responsible for IgG cleavage in wounds and requires an acidic pH for enzymatic activity. As such, we further assessed pSi NPs ability to provide proteolytic protection using a protease elevated in DRFUs; MMP3. MMP3‐induced proteolytic cleavage of antibodies is well‐documented in the literature.^[^ ^85^, ^86^ ^]^ Exposure to MMP3, wash, and a release experiment was performed similarly to above, except the protease incubation did not require an acidic environment, thus better reflecting the DRFU microenvironment. There was some cleavage of FnAb to lower molecular weight fragments at day 1, but the majority of FnAb remained intact over the 4 day trial (Figure 5C). A low level of cleavage is not unexpected as some FnAb would also be bound to the exposed outer surface of pSi NPs, which would not be protected from degradation. Confirming the assay conditions were effectively optimized for MMP3 activity, free FnAb exposed to MMP3 displayed extensive cleavage.

Together, these findings demonstrate pSi NPs to effectively shield encapsulated FnAb from degradation by specific proteases present at high concentrations in DRFUs. As such, pSi NPs are expected to extend the half‐life of FnAb (or other loaded therapeutic antibodies) within these wounds, potentially improving the therapeutic effect by better capturing the therapeutic window.

### 2.6 Thermo‐Sensitive Hydrogel Formulation Facilitates FnAb Release from pSi NPs

**Figure 6:**
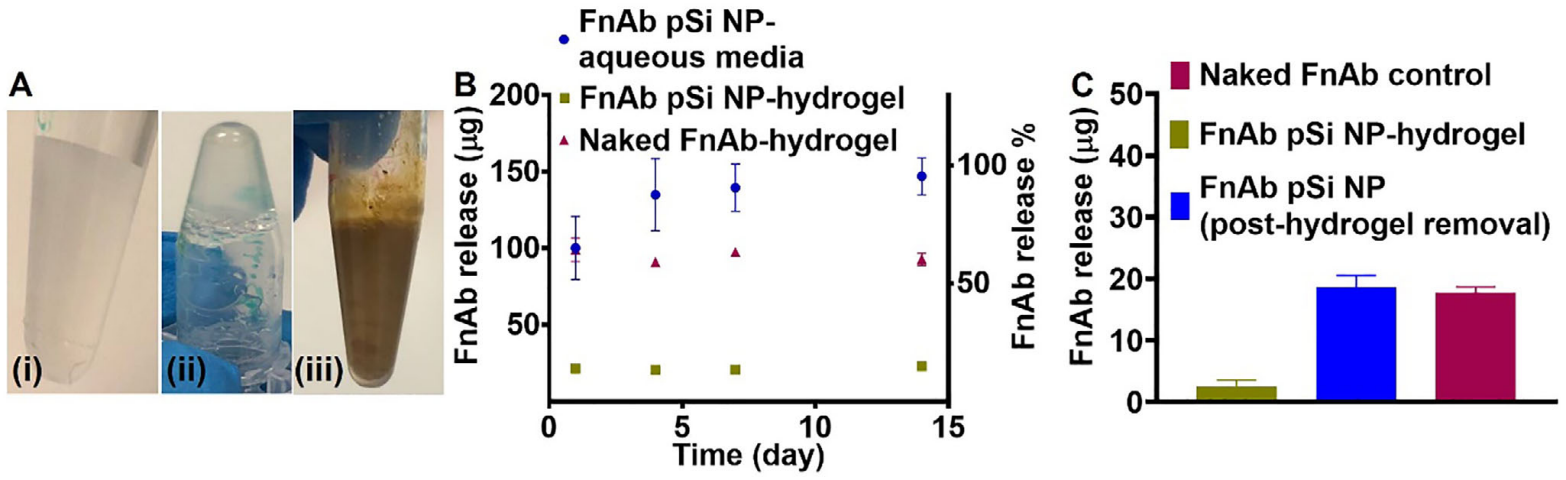
Thermo‐sensitive hydrogel formulation for FnAb pSi NPs. A) Representative photos of the thermo‐sensitive hydrogel used to formulate FnAb pSi NPs for topical administration to wounds. i) Hydrogel in liquid phase (4 °C) and ii) in solid phase (34 °C). iii) FnAb pSi NP hydrogel in liquid phase (4 °C). B) Detection of functional antibody release in FnAb pSi NPs formulated in hydrogel and incubated for 14 days at 34 °C. Control samples include naked FnAb formulated into hydrogel and FnAb pSi NPs in aqueous media (release buffer: Tris, 0.1% Tween 20, 5% (w/v) BSA). C) Detection of functional antibody release in FnAb pSi NPs formulated in hydrogel and incubated for 3 days at 34 °C, then the hydrogel was removed, and a release experiment performed in release buffer for 2 days at 25 °C (blue). Control samples include FnAb pSi NPs formulated into hydrogel (green) and naked FnAb in release buffer (burgundy). Functional FnAb in the release samples (B,C) were quantified by ELISA and presented as mean ± STD (*n* = 3).

To topically deliver FnAb pSi NPs to DRFUs, we opted for the use of a thermo‐sensitive hydrogel formulation. The hydrogel, comprised of 19% (w/v) pluronic F‐127, 10% (v/v) propylene glycol, and 71% (v/v) aqueous phase, forms a liquid gel at 4 °C.^[^ ^87^, ^88^ ^]^ Formulation of FnAb pSi NP (500 µg FnAb and 5 mg pSi) was achieved by addition and simple mixing to create a homogenous solution (300 µL) (**Figure** **6**A). The FnAb pSi NP hydrogel solidifies when applied to and warmed by the skin (sets > ≈30 °C) due to thermo‐sensitive micellar reorganization, consistent with the thermo‐responsive behavior of pluronic F‐127.^[^ ^89^ ^]^ This solidification was designed to retain FnAb pSi NPs at the wound site, but also provide the moisture retention effect associated with Pluronic F‐127^[^ ^87^, ^88^ ^]^ and propylene glycol.^[^ ^90^, ^91^ ^]^

Naked FnAb was formulated into the hydrogel to assess if it damages antibody functionality. As measured by ELISA, there was no reduction in FnAb functionality when incubated for 14 days at 34 °C (i.e., hydrogel in solid state – as it would be on wounds, Figure 6B). When FnAb pSi NPs were formulated into the hydrogel, there was minimal detection of FnAb in the hydrogel over 14 days at 34 °C, and detection did not increase over time, suggesting that FnAb remained entrapped within the pSi NPs with no release into the hydrogel under these solid‐state conditions. Note that the hydrogel only transiently remains solid immediately following topical application onto the wound. Upon contact with the moist wound environment, the hydrogel begins to absorb wound fluid and gradually liquefies, facilitating the release of fully functional FnAb from the pSi NPs directly into the wound bed. This hydrogel absorption commences immediately post‐application, as supported by our in vivo observations and consistent with previous literature.^[^ ^88^ ^]^ This mechanism ensures the therapeutic availability of the FnAb at the wound site, making FnAb pSi hydrogel formulation well‐suited to this clinical wound therapy application.

The lack of FnAb release from pSi NPs in solid hydrogel suggests this formulation may be safely stored under these conditions long‐term. However, confirmation that FnAb released from pSi NPs following hydrogel removal retained its function was required. Therefore, FnAb pSi NPs were incubated in the hydrogel for 3 days at 34 °C, the hydrogel was removed, and then a release experiment was performed for 2 days at 25 °C. To remove the hydrogel, the sample was cooled to 4 °C to allow liquification of the hydrogel, with FnAb pSi NPs pelleted by centrifugation, hydrogel decanted, and the pellet resuspended in release buffer. As expected, FnAb pSi NPs embedded in hydrogel for 3 days exhibited negligible FnAb release as determined by ELISA (Figure 6C). Once the hydrogel was removed, functional FnAb was effectively released (Figure 4C). These findings confirm that the hydrogel matrix successfully retains FnAb within the pSi NPs, but once removed allows functional FnAb release.

### 2.7 FnAb pSi‐NP Hydrogel Improves Wound Healing in Type 1 Diabetic Mice

Wound healing is a multi‐phase process involving hemostasis, inflammation, tissue proliferation, and remodeling.^[^ ^92^ ^]^ In diabetic wounds, healing is impaired due to intrinsic (such as vascular problems, neuropathy, increased inflammation) and extrinsic (such as callus formation, infections) complications.^[^ ^93^, ^94^, ^95^ ^]^ Chronic diabetic wounds are often stalled in the inflammatory phase during the first weeks post‐injury, contributing to impaired progression into tissue regeneration, and delayed or failed DRFU healing.^[^ ^94^, ^95^, ^96^ ^]^ Therapeutic interventions such as sustained delivery of bioactive agents that actively modulate the wound environment within this window can help facilitate the transition to a pro‐healing phenotype and are therefore considered clinically impactful. Hence, we determined that the first two weeks post‐injury represent a critical window for therapeutic intervention to modulate inflammation and promote tissue repair. The FnAb‐loaded pSi NP formulation provided controlled antibody release over approximately two weeks in vitro, well aligning with this critical therapeutic window. The following in vivo experiments were designed to assess the efficacy of our formulation during this biologically and clinically relevant timeframe in both type 1 and type 2 diabetic mouse wound models.

**Figure 7:**
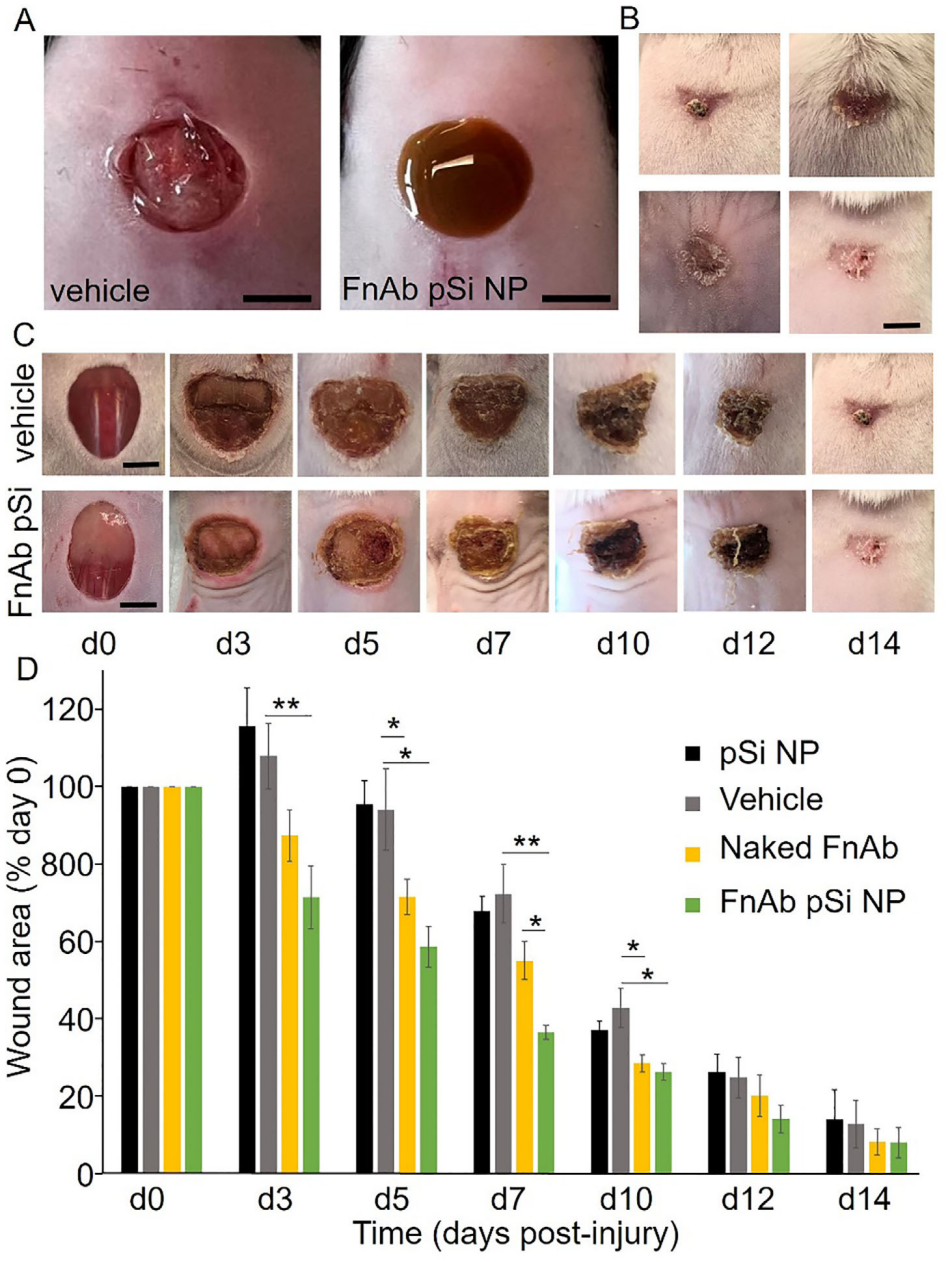
FnAb pSi NP hydrogel improves wound healing in STZ‐induced type 1 diabetic mice. A) Representative images of hydrogel vehicle and FnAb pSi NP hydrogel when applied to wounds. Representative images B,C) and wound area quantification D) of mice wounds treated with FnAb pSi NPs, naked FnAb, pSi, or vehicle control (*n* = 8 mice per group). Wound area is expressed for each group as percentage of day 0 wounds (mean ± SEM). ^*^ *p* <0·05, ^**^ *p* <0.005. Size bars = 5 mm.

In the first trial, FnAb pSi NP hydrogel was administered to single 10 mm diameter excisional wounds in streptozotocin (STZ)‐induced type 1 diabetic mice, involving a single topical application at the time of injury (see Figure S4A, Supporting Information, for experimental flow chart). The FnAb pSi NP topical gel formulation was prepared on ice in its liquid phase, which was then pipetted directly onto the wound where it immediately solidified (see **Figure** **7**A for representative images). There were no differences in weights between the mouse treatment groups, and all mice remained above 90% of the original weight (Figure S4B, Supporting Information). Additionally, blood glucose levels remained above 15 mm throughout the trial, confirming a diabetic phenotype, and there was no difference between the groups (Figure S4C, Supporting Information).

FnAb pSi NP hydrogel‐treated mice displayed a significant improvement in wound closure compared to vehicle‐ and pSi‐treated controls (area under curve (auc), *p* <0.005, *n* = 8 mice per group, Figure 7B–D). The effect of FnAb pSi NPs was most pronounced from day 3 to day 10 post‐injury. Naked FnAb also significantly improved wound closure compared to the vehicle control (auc, *p* = 0.04). However, healing was significantly faster when FnAb was packaged into pSi NPs (auc, *p* = 0.05). There was no difference in wound healing between pSi‐treated and vehicle‐treated mice, suggesting pSi alone had no effect on wound healing.

**Figure 8:**
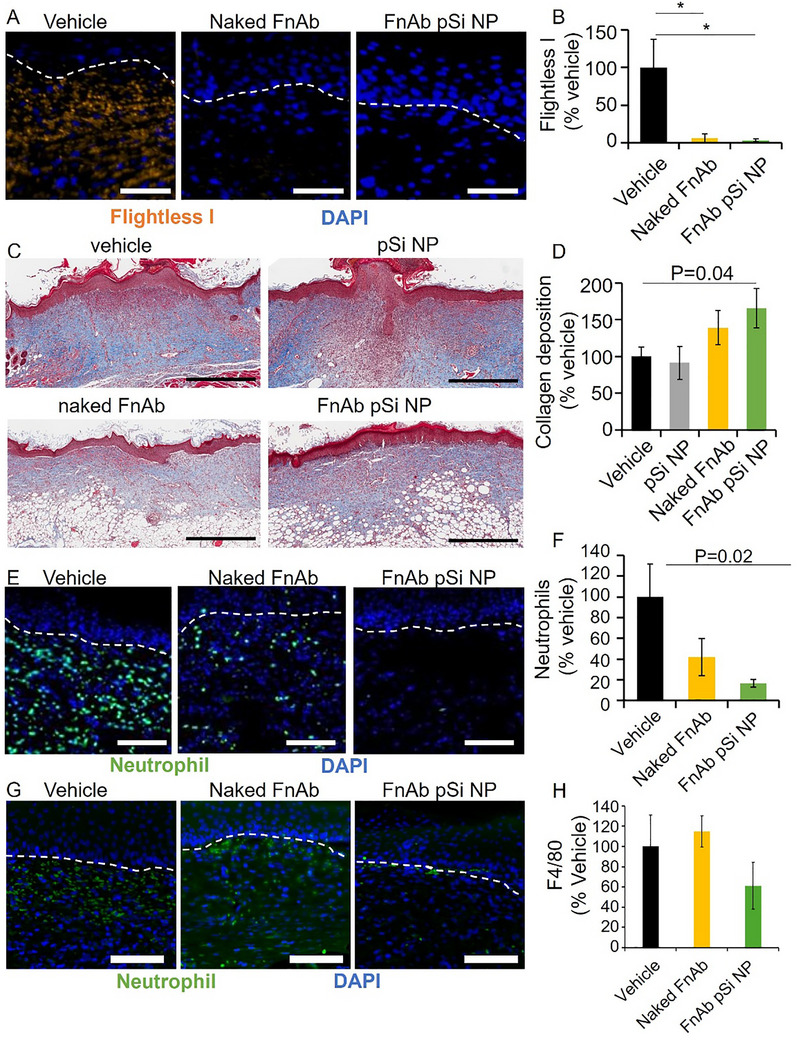
FnAb pSi NPs reduce Flightless I, improve collagen maturation, and decrease inflammation in STZ‐induced type 1 diabetic wounds. A) Representative images of Flightless I immune‐positivity in the granulation tissue of STZ‐induced diabetic mouse wounds at day 14 post‐injury. Samples were assessed in mice treated with FnAb pSi NPs, naked FnAb, or vehicle control (*n* = 4 wounds analyzed per group). Orange = Flightless I. Blue = DAPI nuclear stain. B) Flightless I quantification in mice excisional wounds. Data presented as Flightless I immune positivity as a percentage of vehicle. ^*^ *p* <0·05 compared to vehicle‐treated mice. Bonferroni correction was used for multiple pairwise tests. Size bars = 100 µm. Representative Masson's trichrome stained wound tissue (C) and quantification of mature collagen (D) at day 14 post‐injury. Data presented as collagen deposition as a % of vehicle. Size bars = 700 µm. All pairwise comparisons were analyzed by Student's *t*‐test (two‐sided, nonpaired). Bonferroni correction was used for multiple pairwise tests. Representative images (E) and quantification (F) of neutrophils (NIMP‐R14) present in the granulation tissue of diabetic wounds treated with FnAb pSi NP, naked FnAb, and vehicle. Representative images (G) and quantification (H) of macrophages (F4/80) in db/db diabetic mice wound granulation tissue. Samples are at day 14 post‐injury. Orange = neutrophils. Blue = DAPI nuclear stain. The dashed lines in (A,E) denotes the intersection of the dermis and epidermis. Data in (B,D,F) is presented as mean ± SEM (n≥4 per group). Size bars = 100 µm. All pairwise comparisons were analyzed by Student's *t*‐test (two‐sided, nonpaired). Bonferroni correction was used for multiple pairwise tests.

At the day 14‐post‐injury experimental endpoint, all wounds were >80% closed thus the effect of FnAb‐pSi NP hydrogel on wound area was not as pronounced as earlier in the wound healing process, limiting the use of histology at this endpoint to further quantify wound closure. Histologically at day 14 post‐injury, and agreeing with the macroscopic data, FnAb pSi NP‐treated mice displayed a trend towards improved healing, with reduced wound area and wound gape (non‐significant, Figure S5A–C, Supporting Information). However, immunohistochemistry of wound granulation tissue at day 14 post‐injury identified Flightless I to be significantly reduced in both FnAb pSi NP‐ and naked FnAb‐treated mice (*n* = 4 per group, *p *<0.05, **Figure** **8**A,B). Masson's Trichrome staining of d14 post‐injury wounds showed FnAb pSi NP hydrogel to significantly improve collagen deposition and maturation compared to vehicle‐treated controls (*p* = 0.04, *n* = 8 per group), with naked FnAb having a less pronounced effect (Figure 8C,D).

In mice expressing low levels of Flightless I (i.e., heterozygous), there is reduced neutrophil and macrophage recruitment to wounds, whereas in Flightless I over‐expressing mice (i.e., transgenic), there is increased neutrophil and macrophage recruitment,^[^ ^97^ ^]^ a pattern also observed in type 1 diabetic mouse wounds.^[^ ^52^ ^]^ In response to FnAb pSi NP hydrogel, there were significantly reduced numbers of neutrophils in the wound margin of the type 1 diabetic mice at day 14 post‐injury compared to the vehicle treatment group (*p* = 0.02, n ≥ 3 per group, Figure 8E,F). FnAb pSi NP hydrogel treatment also led to a reduction in macrophages compared to the vehicle treatment group, although the effect was not statistically significant, likely due to the wound being assessed late in the healing cascade (d14 post‐injury, n ≥ 3 per group, Figure 8G,H).

### 2.8 FnAb pSi NPs Hydrogel Improves Wound Healing in db/db Type 2 Diabetic Mice

**Figure 9:**
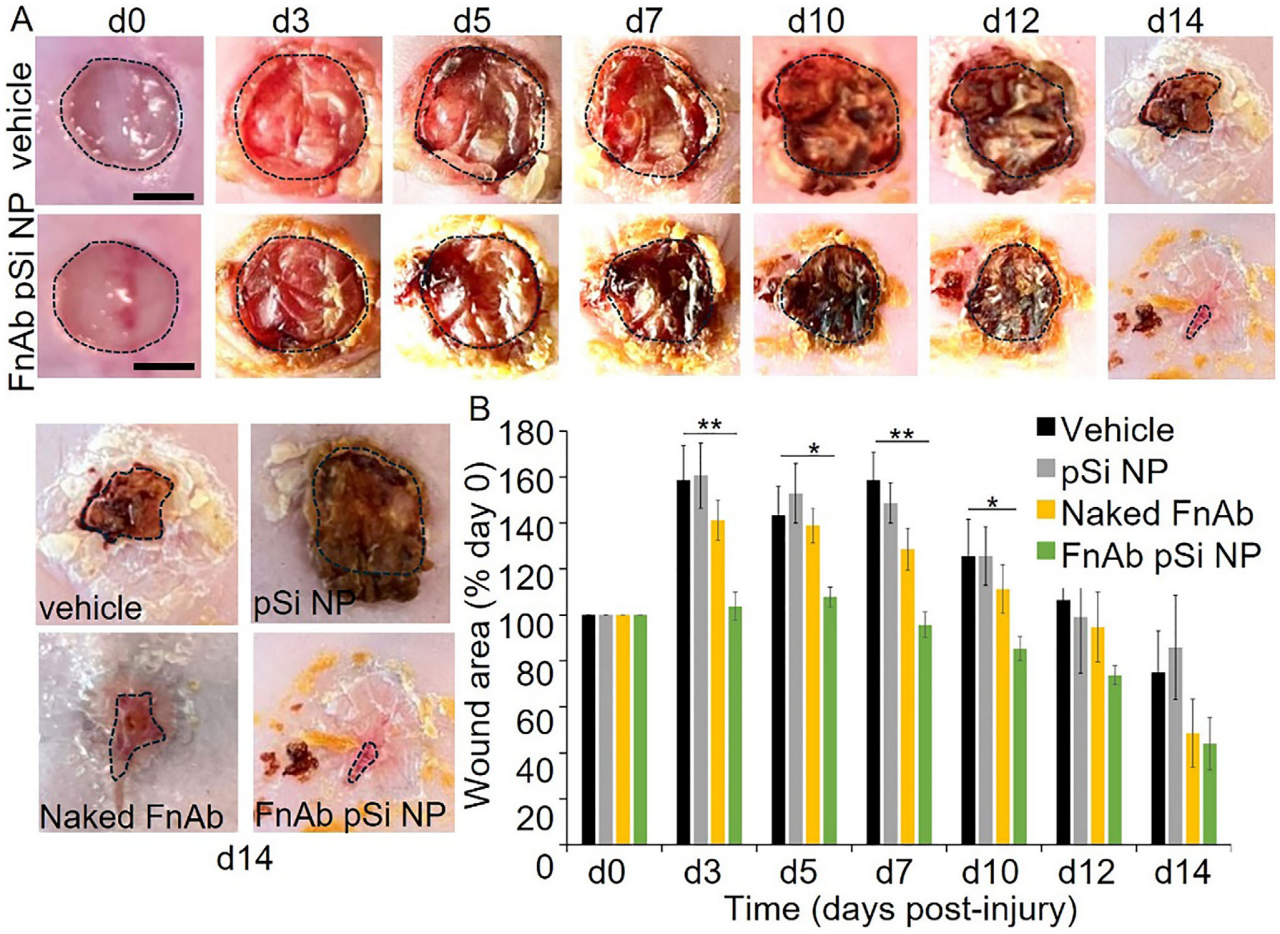
FnAb pSi NP hydrogel improves wound healing in db/db diabetic mice. A) Representative images, B) and size quantification of mice excisional wounds treated topically with FnAb pSi NP, naked FnAb, pSi NP, or vehicle control. Wounds were treated at the time of injury and assessed over 14 days. Data is presented as mean ± SEM (*n* = 8 per group). ^*^ *p* <0·05, ^**^ *p* <0.005. Size bar = 5 mm. All pairwise comparisons were analyzed by Student's *t*‐test (two‐sided, nonpaired). Bonferroni correction was used for multiple pairwise tests.

**Figure 10:**
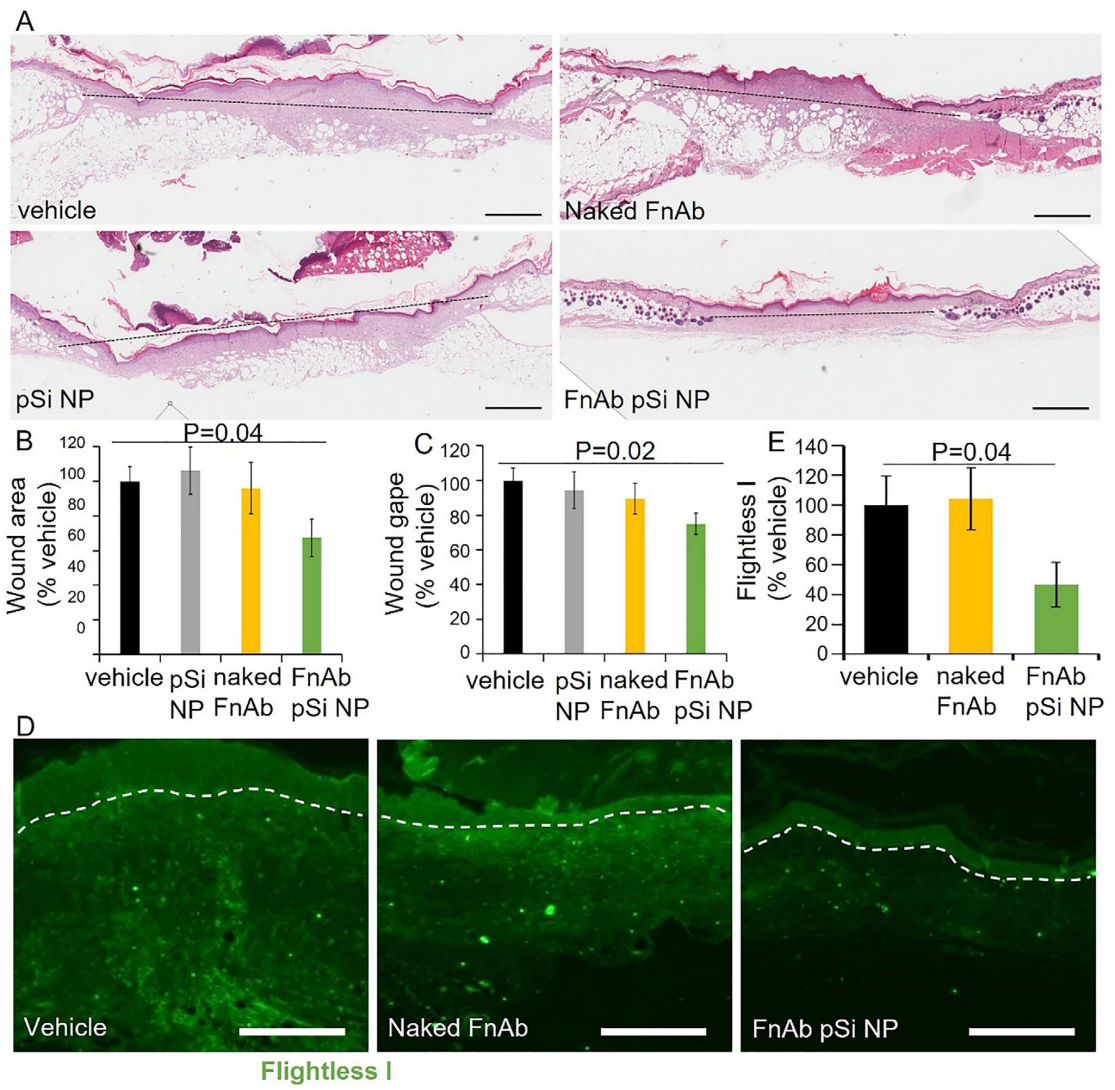
FnAb pSi NP hydrogel improves wound gape, area, and reduces detection in db/db diabetic mice. Representative H&E stained tissue section images (A), quantification of wound area (B), and gape (C) of FnAb pSi NP‐treated excisional wounds at day 14 post‐injury. Black dashed lines indicates wound gape. Representative Flightless I images (D) and quantification (E) in FnAb pSi NP‐treated excisional wounds at day 14 post‐injury. Data presented as a percentage of vehicle, mean ± SEM (*n* = 8 per group). White dashed lines represent the dermal‐epidermal junction. Size bars in (A) = 1 mm and D = 250 µm. All pairwise comparisons were analyzed by Student's *t*‐test (two‐sided, nonpaired). Bonferroni correction was used when multiple pairwise tests were performed.

A second trial to further assess FnAb pSi NP hydrogel was performed, this time in db/db mice, a type 2 diabetes model better reflecting the patient population most commonly experiencing DRFUs. These mice have a more pronounced impairment in wound healing than STZ‐induced diabetic mice and are considered a more clinically relevant model to assess the wound healing properties of FnAb pSi NP hydrogel.^[^ ^98^, ^99^, ^100^, ^101^ ^]^ As with the STZ‐diabetic induced mice, the treatment was well tolerated, with no adverse events or excess weight loss identified (Figure S6, Supporting Information). The wound area of FnAb pSi NP‐treated wounds was significantly reduced compared to the vehicle‐treated and pSi‐treated controls (auc, *p* <0.005, **Figure** **9**A,B). Naked FnAb also significantly improved wound closure (auc, *p *<0.05) but the FnAb pSi NP treatment was significantly better than this treatment group (auc, *p* <0.05). Histologically, wound area (*p* = 0.04) and gape (*p* = 0.02) were significantly reduced in the FnAb pSi NP treated compared to vehicle‐treated controls (**Figure** **10**A–C).

**Figure 11:**
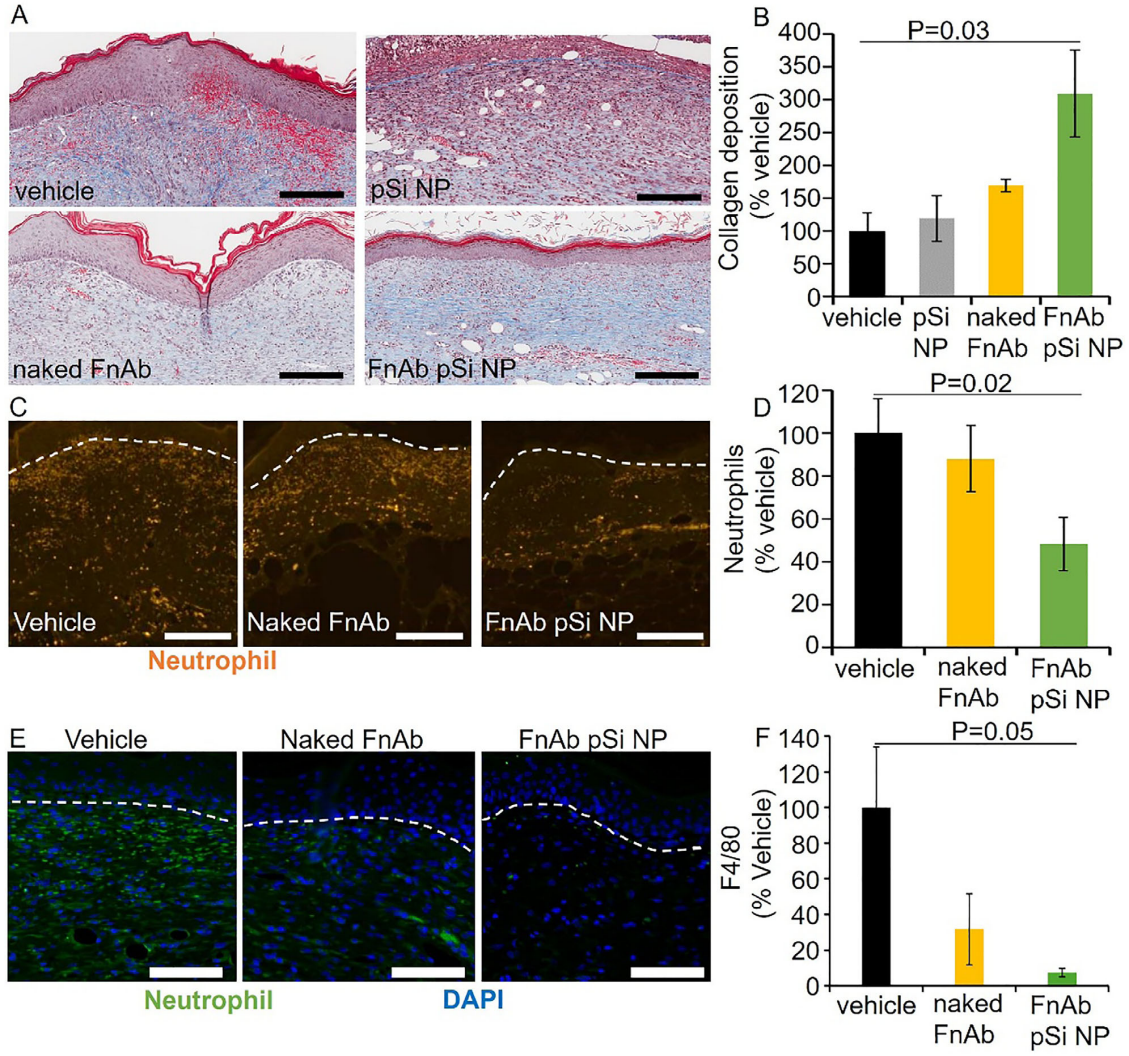
Improved collagen maturation and reduced inflammation in FnAb pSi NP‐treated db/db diabetic mice. Representative Masson's trichrome‐stained wound tissue (A) and quantification of mature collagen (B) at day 14 post‐injury. Presented as collagen deposition as a % of vehicle. Representative images (C) and quantification (D) of neutrophils (NIMP‐R14) in db/db diabetic mice wound granulation tissue at 14 days post‐injury. Representative images (E) and quantification (F) of macrophages (F4/80) in db/db diabetic mice wound granulation tissue at 14 days post‐injury. Dashed lines represent the dermal‐epidermal junction. Data presented as mean ± SEM (n ≥4 per group). Size bars = 600 µm (A), 250 µm (C), and 100 µm (E). All pairwise comparisons were analyzed by Student's *t*‐test (two‐sided, nonpaired, *n* = 4 per group).

Flightless I was significantly reduced in FnAb pSi NP‐ but not naked FnAb‐treated mice compared to the vehicle‐treated control (*p* = 0.04, Figure 10D,E). Masson's Trichrome staining of d14 post‐injury wounds showed FnAb pSi NP hydrogel to significantly improve collagen deposition and maturation compared to vehicle‐treated controls (*p* = 0.03, *n* = 8 per group, **Figure** **11**A,B). Neutrophils were significantly reduced in FnAb pSi NP‐ but not naked FnAb‐treated mice compared to the vehicle‐treated control (*p* = 0.02, Figure 11C,D). FnAb pSi NP hydrogel treatment also led to a reduction in macrophages compared to the vehicle treatment group (*p* = 0.05, n ≥ 3 per group, Figure 11E,F). FnAb pSi NP‐hydrogel therefore had a similar dampening effect on inflammation in both type I and type II diabetic wounds. Together, pSi NP hydrogel is an effective tool to deliver FnAb to diabetic wounds, leading to faster and more mature healing, with reduced inflammation. FnAb pSi NP‐hydrogel is more effective than naked FnAb hydrogel, and displays no evidence of unwanted side effects.

## 3 Conclusion

We developed a formulation to effectively deliver therapeutic antibodies to DRFUs. Packaging the wound healing therapeutic FnAb into pSi NPs afforded protection from proteases present in diabetic wounds and overcame a wound environment found to damage administered therapeutics. The functional antibody was released in a controlled fashion from pSi NPs, thereby allowing effective delivery of the antibodies, helping facilitate the transition of diabetic wounds from a pro‐inflammatory to a pro‐healing phenotype. Untreated db/db diabetic mice wounds healed at a slower rate than STZ‐induced diabetic wounds, yet the effect of FnAb pSi NPs was more pronounced, further highlighting the benefit of this delivery system in diabetic and other chronic wound environments. The formulation of FnAb pSi NP into the thermo‐sensitive hydrogel improved delivery of the antibody to the wound, which would be directly translatable to the clinic, potentially as an easy‐to‐administer treatment over‐the‐counter option. Notably, Pluronic F‐127 is both biocompatible and Food and Drug Administration (FDA)‐approved.^[^ ^102^ ^]^ Controlled release was observed over 2 weeks, with most antibody released within the first 7 days, making it ideally suited for clinical application, as this coincides with routine dressing changes. Our pSi NP preparations have been specifically tailored for FnAb loading and release but could be easily adapted for the delivery of other therapeutic antibodies. FnAb pSi NP hydrogel is therefore an exciting and clinic‐ready therapeutic approach for the treatment of DRFUs. Together, these findings introduce a novel therapeutic strategy by targeting cytoskeletal remodeling through antibody‐mediated inflammatory modulation in diabetic wounds. The advanced delivery platform‐biodegradable pSi NPs embedded in a clinically approved thermo‐responsive hydrogel‐achieves sustained, protease‐shielded antibody release over 14 days. With direct topical applicability and translational readiness, this formulation represents a promising and impactful approach to address the critical unmet clinical need in diabetic wound healing.

## 4 Experimental Section

### Materials

Hydrofluoric acid (HF) (48% Merck) and ethanol (Ajax, absolute, 100%) were used without further purification. Milli‐Q water was used from an Advantage A10 water purification system (Merck Millipore, Q‐POD, water resistivity of 18.2 m Ω cm at 25 °C, TOC < 5 ppb). SIGMAFAST o‐phenylenediamine dihydrochloride (OPD) substrate, pepsin from porcine gastric mucosa (97.5%), STZ, BSA, Tris buffered saline, Tris buffered saline with Tween, sodium acetate trihydrate, and Dulbecco's phosphate buffered saline (PBS) solution were purchased from Sigma–Aldrich. FnAb was provided by AbRegen (Adelaide, Australia). Recombinant Flightless I protein (partial sequence; 495‐827 aa,) was obtained from Cusabio Technology LLC (Houston, TX, USA). Donkey anti‐rabbit IgG (H+L) Horseradish Peroxidase (HRP) and Alexafluor 488/594‐conjugated secondary antibodies were from Invitrogen (Carlsbad, CA, USA). Polyclonal goat anti‐mouse horseradish peroxidase, murine non‐specific IgG antibody (I8765), Corning 96‐well Clear Flat Bottom Polystyrene High Bind Microplate, Pluronic F‐127, propylene glycol, Sera human (frozen human serum), ɑ‐Chymotrypsin (from bovine pancreas) were bought from Sigma–Aldrich. rhMMP3 was provided by R&D systems (Minneapolis, MN, USA). Human plasma was from Cayman Chemical (Ann Arbor, Michigan, USA). Human Flightless I monoclonal antibody (3G2) was from GroPep Bioreagents (South Australia, Australia). Primary antibody against Flightless I (PA5‐82369) and phenylmethyl sulfonyl fluoride (PMSF) were purchased from ThermoFisher Scientific (Victoria, Australia). Neutrophil marker antibody (NIMP‐R14, sc‐59338) was from Santa Cruz (Texas, USA). Mini‐PROTEAN TGX Stain‐Free 4‐15% Protein Gels from BIO‐RAD (USA) were used. Boron‐doped (0.00055–0.001 Ω cm resistivity) silicon wafers (<100> orientation) were from Siltronix, France.

### Fabrication and Surface Modification of pSi NPs

A range of nanocarriers was prepared for downstream testing, including those with different surface chemistries. FEG‐SEM (FEI Nova NanoSEM 430) and HR‐TEM (FEI Tecnai G2 T20 TWIN) imaging were used to characterize pSi particle size and pore dimensions.

A two‐step electrochemical etching process applied via a wet‐bench setup was used to produce pSi from boron‐doped (0.00055–0.001 Ω cm resistivity) silicon (Si) wafers (<100> orientation, Siltronix, France), as described in the following. The Si wafer underwent anodization in a 3:1 (v/v) hydrofluoric acid (HF):ethanol solution employing an alternating current profile (50 mA cm^−2^ for 4.3 s, followed by 188 mA cm^−2^ for 0.2 s) repeated for 84 min. This yielded a pSi film exhibiting alternating high and low porosity layers, known as NP layers and perforation layers, respectively. Electropolishing (1:1 HF:ethanol, 188 mA cm^−2^ for 90 s) detached the etched film from the silicon substrate.

Subsequently, two methods were used to generate surface‐oxidized pSi NPs. In the first method, pSi films were sonicated in DMSO for 16 h, facilitating the generation of chemically oxidized pSi NPs. In the other method, pSi films were first thermally oxidized (200 °C, 2 h) in a tubular furnace, and subsequently were sonicated in absolute ethanol for 20 h.

Sonication of the pSi films led to the production of pSi particles of varying sizes. Size selection was achieved by two‐step centrifugation. First, the pSi particle solution was centrifuged at 1800 rcf for 8 min to collect the supernatant. Then, the supernatant was centrifuged at 20 000 rcf for 10 min to collect a pellet of uniformly sized pSi NPs. This two‐step process was repeated three times, which removed both excessively large and small particles, resulting in a stable suspension of reasonably monodisperse pSi NPs.

### Antibody Loading Into pSi NP

To load FnAb into oxidized pSi NPs (FnAb‐pSi NPs), 10 mg pSi NPs were mixed with FnAb (1 mg mL^−1^) in sealed, low‐protein‐binding Eppendorf tubes and incubated at 4 °C for 20 h. Antibody‐loaded pSi NPs were pelleted by centrifugation (10 min at 20000 rcf). Unbound Antibody was removed by a subsequent centrifugation (10 min at 20 000 rcf) after replacing the supernatant with fresh PBS (pH 7.4).

To further investigate the effect of pH on FnAb loading into pSi NPs, an additional experiment was performed using three buffer conditions: pH 6 (15 mm acetate buffer, adjusted with 0.1 m HCl), pH 7.4 (PBS), and pH 8.0 (20 mm Tris buffer, adjusted with 0.1 m NaOH). All buffers were prepared at low ionic strength to minimize potential disruption to antibody structure and to maintain antibody stability across the tested pH range. In each condition, 10 mg of pSi NPs were incubated with 1 mg FnAb (1 mg mL^−1^) in sealed, low‐protein‐binding Eppendorf tubes at 4 °C for 20 h. After incubation, unbound antibody was removed by centrifugation and the pellet was washed once with a fresh buffer to remove loosely bound antibody.

Antibody loading was quantified using a Pierce BCA assay (ThermoFisher Scientific, following the manufacturer's protocol) and by measuring supernatant absorbance at 562 nm via UV–vis spectrophotometry (NanoDrop 2000, Thermo Scientific) before and after incubation with pSi NPs. Antibody loading per mg pSi was calculated using a standard curve. Data were presented as the mean ± SD. Statistical analyses were performed using a *t*‐test for comparison between two groups and a one‐way ANOVA for comparisons among three groups to assess differences across pH conditions. Statistical significance was defined as p < 0.05. The antibody‐loaded pSi NPs were used for subsequent experiments as described below.

### In Vitro Antibody Release Test

FnAb release from prepared FnAb‐loaded pSi NPs was assessed under two conditions: (i) incubation in 300 µL of Tris buffer containing 0.1% Tween 20 and 5% (w/v) BSA; and (ii) incubation in 300 µL of a SWF (1:1 mixture of human plasma and human serum). Experiments were conducted at 25 °C and 34 °C. Release samples were collected at 1, 4, 7, and 14 days by centrifuging pSi NPs at 20000 rcf for 10 min to obtain the supernatant. The removed supernatant was replaced with an equal volume of fresh medium. The release samples were quantified using an ELISA assay described below. The data obtained from release tests were normalized against a standard curve generated from serial dilutions of the antibodies.

FnAb concentrations were determined by an indirect ELISA (Figure S3, Supporting Information). High‐bind clear flat‐bottom 96‐well microplates were coated with 50 µL well^−1^ of recombinant Flightless I protein (partial sequence, 10 µg mL^−1^ in 0.1 m NaHCO_3_, pH 8.5) and incubated overnight at 4 °C. After discarding unbound protein and washing five times with Tris buffer and blocking with 5% (w/v) BSA for 4 h at room temperature, 200 µL well^−1^ of supernatants (pre‐cleared of pSi NPs by centrifugation at 20000 rcf for 10 min) and FnAb standards (fresh thawed aliquots, 0.01–128 µg mL^−1^) were added and incubated overnight at 4 °C. Subsequently, plates were incubated with 100 µL of peroxidase‐conjugated goat anti‐mouse IgG secondary antibody (1:400 dilution) for 1 h at room temperature, washed five times with Tris buffer, and developed with OPD, a chromogenic substrate (100 µL well^−1^) for 20 min at room temperature. Absorbance was measured at 410 nm using an automated microplate reader (BioTek, SYNE GYMx). FnAb concentrations were calculated by interpolation from the standard curve.

Following the release experiments, the cumulative release data obtained at 34 °C (as this temperature was more clinically relevant) were fitted to several commonly used mathematical models including zero‐order, first‐order, Higuchi, and Korsmeyer‐Peppas models to determine the potential release mechanisms.^[^ ^25^ ^]^ An overview of the mathematical release kinetics models and their parameters is provided in Table S2 (Supporting Information). In all models, m_t_ represents the cumulative amount of antibody released at time t, while m_0_​ refers to the initial amount of antibody present in the formulation. k_0_ and k_1_ ​ are the release rate constants for the zero‐order and first‐order models, respectively. For the Korsmeyer‐Peppas model, m_∞_ denotes the total amount of drug released at infinite time, k is the release rate constant, and n is the release exponent that provides insight into the underlying release mechanism. In this model, n ≤ 0.43 indicates Fickian diffusion (i.e., drug release is primarily controlled by diffusion), and 0.43 < n < 0.85 suggests non‐Fickian (anomalous) transport (i.e., a combination of mechanisms such as diffusion and particle degradation). Each model was fitted to the release data and the accuracy of the fit was assessed using the coefficient of determination (R^2^). For the Korsmeyer‐Peppas model, the diffusional exponent (n) was also calculated. All fitting analyses were performed using GraphPad Prism version 8.2.1.

### Characterization of pSi NP Degradation in SWF

pSi NPs were incubated in 200 µL SWF at 34 °C for 14 days. Samples for TEM were collected at 2 and 14 days as follows. Following a centrifugation to pellet the pSi NPs, the supernatant was discarded, and the pellet was rinsed sequentially with Milli‐Q water:ethanol mixtures (1:0, 2:1, 1:1, 1:2, 0:1, v/v). The pSi NPs were then diluted to 0.1 µg mL^−1^ in ethanol and deposited onto TEM grids before imaging them using high resolution‐TEM (FEI Tecnai G2 T20 TWIN) (Figure S2, Supporting Information).

### Assessing the Antibody Protective Effect of pSi NPs Against Proteolytic Enzymes—Using Pepsin

In this test, the structural integrity of FnAb pSi NPs was assessed in a pepsin‐treated medium as follows. 500 µg FnAb pSi NPs were transiently incubated in 3 µg pepsin‐containing HCl medium with pH 3.3 (200 µL, 0.4 mm) for 1.5 h at 34 °C. As a control, an FnAb pSi NP sample was incubated in the same medium in the absence of pepsin. Next, the medium was discarded, and FnAb pSi NPs were rinsed where they were resuspended in 600 µL phosphate buffer, pH 7.4, mixed well, then re‐spun thrice and spun down and supernatant discarded to remove residual pepsin. Subsequently, pSi NPs were resuspended in 150 µL release buffer (Tris buffer containing 5% (w/v) BSA). The release experiment was performed at 25 °C, with antibody‐containing supernatants decanted at various time points (day 1, day 4, and day 7) for analysis of structural integrity. At each time point, the supernatant was collected and replenished with an equal volume of fresh medium. The collected samples were stored at (−20 °C) then analyzed by SDS‐PAGE. For SDS‐PAGE analysis, conditions including: non‐reducing (no antibody fragmentation), non‐denaturing (no pre‐heating), and Stain‐Free Mini‐PROTEAN Protein Gels (BIO‐RAD) were used for gel electrophoresis (using Mini‐PROTEAN Tetra System). The collected release samples were diluted 1:100 in MQ water. Electrophoresis was performed using 200 V for 30 min. The imaging of gels was performed using ChemiDoc MP Imaging System (Bio‐Rad).

In another test, 100 µg FnAb in loaded pSi NPs were transiently incubated in 400 µL 0.0004 m HCl (pH 3.3) in the presence or absence of pepsin (3 µg) at 34 °C for 90 min. Afterwards, pSi NPs were spun down and pepsin‐containing supernatant was discarded. Then, pSi NPs were rinsed with PBS (3X) to remove residual pepsin, and next, release was continued in the release buffer (Tris buffer, pH 7.4, 5% (w/v) BSA, 25 °C). At day 1, pSi NPs were centrifuged down and the resulting supernatants, designated as 1X pepsin (1XP), were collected. One set of pSi NPs underwent a second incubation with pepsin‐containing medium (pH 3.3) for 90 min, then rinsing in PBS, and the release was continued in the release buffer (hereafter referred to as 2X pepsin (2XP)). The other set of pSi NPs, previously incubated once with pepsin (1XP)), had release continued in the release buffer at 25 °C. On day 3, supernatants from pSi NPs incubated with 2XP and 1XP were collected. One set of pSi NPs underwent a third incubation with pepsin (pH 3.3) for 90 min (hereafter referred to as 3Xpepsin (3XP). Following rinsing, release continued in the release buffer. The other set of pSi NPs (1XP and 2XP) were incubated in the release buffer and release was continued at 25 °C and release was continued. On day 7, supernatants were collected from all pSi NP samples (1XP, 2XP, and 3XP).

### Assessing the Antibody Protective Effect of pSi NPs Against Proteolytic Enzymes—Using MMP3

The assay compounds and protocols were used as per the kit instructions. Assay compounds for this test were prepared with the concentrations as described in the following: Assay buffer (50 mm Tris, 10 mm CaCl_2_, 15 mm NaCl‐ 0.05% Brij 35), MMP3 (10 µg/ 66.7 µL), chymotrypsin (1 mg mL^−1^ in 1 mm HCl), 10 mm PMSF (1.74 mg mL^−1^ in isopropanol), substrate (FnAb antibody). Next, MMP3 was activated via the activation protocol as below. 20 µg mL^−1^ MMP3 were incubated in the assay buffer containing 5 µg mL^−1^ chymotrypsin for 30 min at 37 °C. Then, MMP3 activation was stopped with 2 mm PMSF (pre‐warmed to 37 °C prior to adding to sample). As the next step, 50 µL MMP3‐containing solution were mixed with free FnAb and FnAb‐loaded pSi NPs at a FnAb:MMP3 ratio of 1:1 for 90 min at 34 °C. Regarding the FnAb‐loaded pSi NPs sample, after 90 min, the MMP3‐containing medium was discarded. The FnAb‐loaded pSi NPs were rinsed thrice with Tris buffer to remove residual MMP3, and then resuspended in 60 µL release buffer (Tris, 0.1% Tween 20, pH 7.4). Subsequently, release antibody‐containing supernatants (release medium) were decanted at various time points for analysis of structural integrity. The supernatant was collected at day 1 and replenished with an equal volume of fresh medium, then collected again at day 4. The collected release samples were stored at −20 °C until post analysis by SDS‐PAGE under the following conditions: non‐reducing, non‐denaturing, and undiluted.

### Integrating pSi NPs with a Topically‐Administrable Hydrogel and In Vitro FnAb Release Testing

Topical hydrogel was formulated by mixing 19% (w/v) pluronic F‐127, 10% (v/v) propylene glycol, and 71% (v/v) aqueous phase. The mixture was stored at 4 °C until a homogeneous viscous hydrogel formed. Then, 5 mg of FnAb pSi NPs were dispersed in 300 µL of this hydrogel with gentle mixing to ensure uniform NP distribution. Tris buffer supplemented with 0.1% (v/v) Tween 20 and 5% (w/v) BSA was used as aqueous phase for the following in vitro release test. In vitro FnAb release studies were then conducted at 34 °C. To collect release samples, pSi NP hydrogel samples were cooled to 4 °C and centrifuged (20000 rcf at 4 °C for 60 min) at 1, 4, 7, and 14 days. The supernatant was then carefully removed, replaced with an equal volume of fresh hydrogel, and the pSi NPs were resuspended at 4 °C before resuming incubation at the experimental temperature (34 °C). Release samples (1:100 dilution in Tris buffer (0.1% Tween 20, 5% (w/v) BSA)) were quantified by ELISA; data were normalized to a standard curve of serially diluted FnAb (data presented as mean ± STD (*n* = 3)).

### In Vivo Studies—Animals

BALB/c mice were provided by Monash Animal Research Platform's (MARP) animal breeding facility. Db/db mice were from db/db breeding colony (genetically modified, Leprdb/J #000697, Jackson Laboratory) under Monash Approved ethics number 27334 and bred in Monash Animal Breeding Facility.

### Animal Study Approvals

All animal studies were approved by Monash University (Approval number 37686) and Animal Care and Use Review Office (ACURO) at the USAMRDC Office of Research Protections. All experiments were performed in accordance with all relevant institutional guidelines and regulations. All methods were also reported in accordance with ARRIVE guidelines.

### STZ Induction Of Diabetes

Type 1 diabetes was induced in BALB/c mice via intraperitoneal injection of STZ. At 8‐11 weeks of age, mice received one daily dose of 50 mg kg^−1^ STZ intraperitoneal (dissolved in citrate buffer) for five consecutive days. At 7 and 14 days post‐final STZ injection, blood glucose level (BGL) was evaluated from tail vein prick using glucose strips and a glucometer. Blood glucose levels were measured in the two weeks of the study before wounding to confirm diabetes development (>15 mmol L^−1^). Using this regimen, mice were maintained/monitored for a total of 6 weeks post‐first STZ injection. This allowed time for diabetes to develop, but also, for mice to have diabetes for a prolonged time, such that a delayed healing phenotype develops. Mice maintaining a BGL >15 mm for a minimum of 2 weeks were classified as diabetic, with all others excluded from the trial.

### Excisional Wound Murine Model

Hair was removed from the mouse back by shaving then application of hair removal cream (Veet). A single excisional wound (in the middle of the back (thoraco‐lumbar region)) was created using a 10 mm biopsy punch. Wounds were allowed to heal for 14 days by secondary intention. Wounds photographs were captured throughout the trial for macroscopic analysis. Wound area was calculated as percentage wound area compared to area at day 0 post‐injury (mean ± SEM).

### FnAb pSi NP Administration

FnAb was topically administered only once at the time of injury. Wounds were treated with FnAb pSi NPs (100 µg FnAb loaded into 1 mg pSi NP and then formulated into 200 µL hydrogel), naked FnAb (100 µg FnAb in 200 µL hydrogel), pSi (1 mg unloaded pSi NP formulated into 200 µL hydrogel), and vehicle (200 µL hydrogel) (*n* = 8 mice per group). The thermo‐sensitive hydrogel formulation was 19% (w/v) Pluronic F‐127, 10% (v/v) propylene glycol, and 71% (v/v) aqueous phase. *n* = 8 mice were used per treatment group.

### Morphometric Analysis

Wound gape in the H&E stained slides was a measure of the distance from the undamaged fat layer at each side of the wound. Flightless I was assessed as the staining intensity per area in the wound. Masson's trichrome was assessed as the ratio of old (blue)/new (red) collagen deposited within the wound. Neutrophils were assessed as the number of immune positive cells per area. Slides were blinded before analysis, with at least four images per treatment group analyzed using ImageScope (Leica Biosystems, Wetzlar, Germany). All analysis was performed in wounds at day 14 post‐injury.

### Immunofluorescence

Paraffin‐embedded mouse skin were sectioned at 4 µm then subjected to antigen retrieval using citrate buffer, pH 4 for 10 min at 90 °C, then trypsin digested for 3 min at 37 °C (250 µg mL^−1^). Samples were blocked in PBS, pH 7.2 containing 10% (v/v) goat serum for 30 mins at RT. Primary antibodies against Flightless I (5 µg mL^−1^, PA5‐82369, ThermoFisher Scientific, Victoria Australia), NIMP‐R14 (sc‐59338, 0.5 µg mL^−1^, neutrophil marker, Santa Cruz, Texas, USA) and F4/80 (MCA97R, 5 µg mL^−1^, macrophage marker, BioRad, Hercules, CA, USA), were diluted in 10% (v/v) block, applied to the tissue sections and incubated for 18 h at 4 °C. Species‐specific, Alexafluor 488 or 594‐conjugated secondary antibodies (5 µg mL^−1^; Invitrogen, Carlsbad, CA, USA) were diluted in 10% (v/v) block, incubated for 1 h, and used for primary antibody detection. As a nuclear counterstain, 4,6‐diamidino‐2‐phenylindole (DAPI) was used. Images were captured on an Olympus IX81 microscope and analyzed using AnalySIS software package (Soft Imaging System GmbH, Muenster, Germany).

### Quantification and Statistical Analysis

A minimum of eight mice were included per treatment group in the animal studies. This was based on power calculations assuming a 20% clinically relevant improvement in wound closure, to achieve a statistical significance of *p* = 0·05. Data was analysed for normality using the D'Agostino–Pearson test using a significance level of *p* = 0·05. On rejection, a Mann–Whitney U‐test was performed. For data with a normal distribution, pairwise comparisons were done using Student's *t*‐test (two‐sided, nonpaired). Bonferroni correction was used when multiple pairwise tests were performed on a single set of data. Data were analysed with GraphPad Prism (La Jolla, CA, USA).

## Conflict of Interest

A.J.C. is cofounder of AbRegen and has patents linked to FnAb in this work. N.H.V., A.J.C., and C.T.T. have a patent linked to pSi for drug delivery. All other authors declare that they have no competing interests.

## Supporting information



Supporting Information

## Data Availability

The data that support the findings of this study are available in the supplementary material of this article.
