# Supplementary material for: Porous Silicon Nanoparticle Hydrogel Formulation for Topical Delivery of Flightless I Neutralizing Antibody and Improved Diabetic Wound Healing
Source: Small. 2025 Aug 27;21(41):e05359. doi: 10.1002/smll.202505359 (PMC12530027; doi:10.1002/smll.202505359)
Supplement: Supplementary file 1 — Supporting Information [file SMLL-21-e05359-s001.docx]

Supporting Information

Porous Silicon Nanoparticle Hydrogel Formulation for Topical Delivery of Flightless I Neutralizing Antibody and Improved Diabetic Wound Healing

Christopher T. Turner,^1,2,3&^ Parham Sahandi Zangabad,^1,2&^ Kiralee Janusaitis,^3^ Nikki Black,^3^ Robert Fitridge,^4^ Allison J. Cowin,^3^* Nicolas H. Voelcker^1,2,5^*

1. Drug Delivery, Disposition and Dynamics, Monash Institute of Pharmaceutics Science, Monash University, Parkville Campus, Parkville, VIC, 3052, Australia
2. Melbourne Centre for Nanofabrication, Victorian Node of the Australian National Fabrication Facility, Clayton, VIC 3168, Australia
3. Regenerative Medicine, Future Industries Institute, University of South Australia, Adelaide, SA 5095, Australia
4. Faculty of Health and Medical Sciences, University of Adelaide, Adelaide, SA 5005, Australia
5. Department of Materials Science and Engineering, Monash University, Clayton, VIC 3800, Australia

^&^ These authors contributed equally to this work.

* Corresponding authors: [Nicolas.Voelcker@monash.edu](mailto:Nicolas.Voelcker@monash.edu), [Allison.Cowin@unisa.edu.au](mailto:Allison.Cowin@unisa.edu.au)


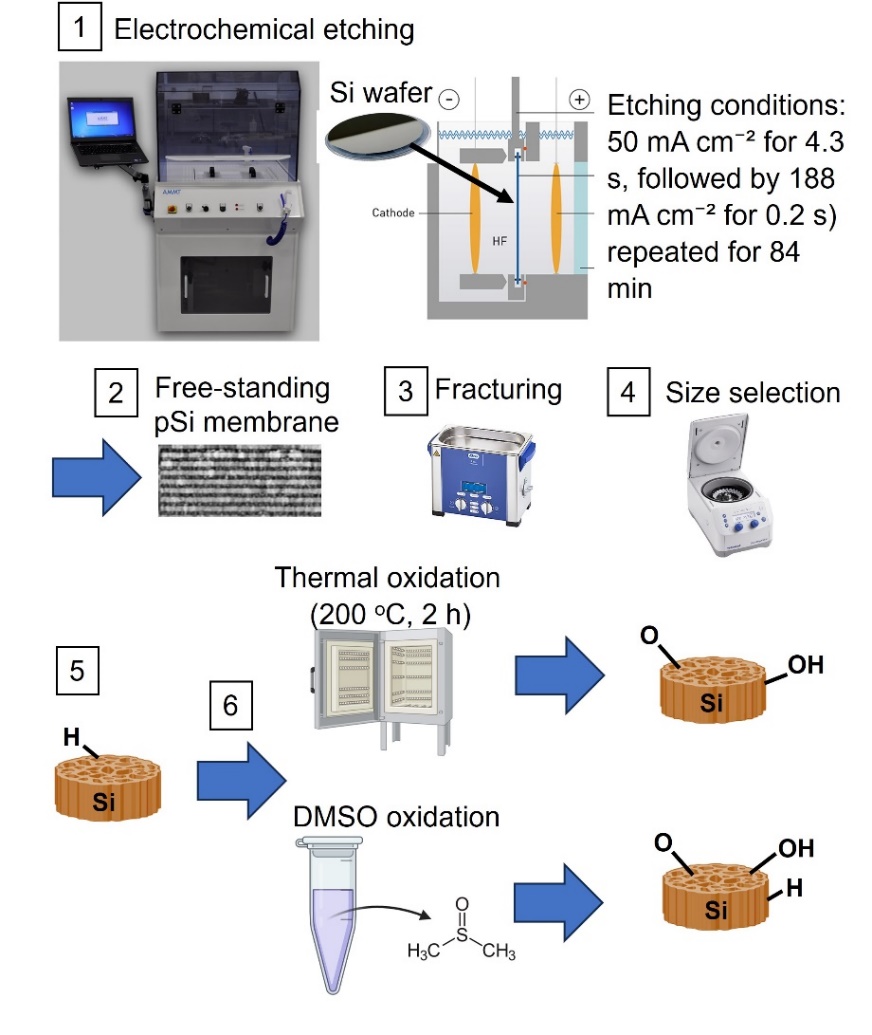


**Figure S1. pSi NP fabrication and surface modification.** pSi NP was fabricated by electrochemical etching of silicon wafers (1). Etched pSi membranes were lifted off from silicon wafer (2) and broken down into particles of various sizes using sonication (3). Optimal particle size range was then selected (4). pSi surface (5) was modified to optimize antibody loading and release via thermal oxidation (using a furnace) or DMSO oxidation (6).


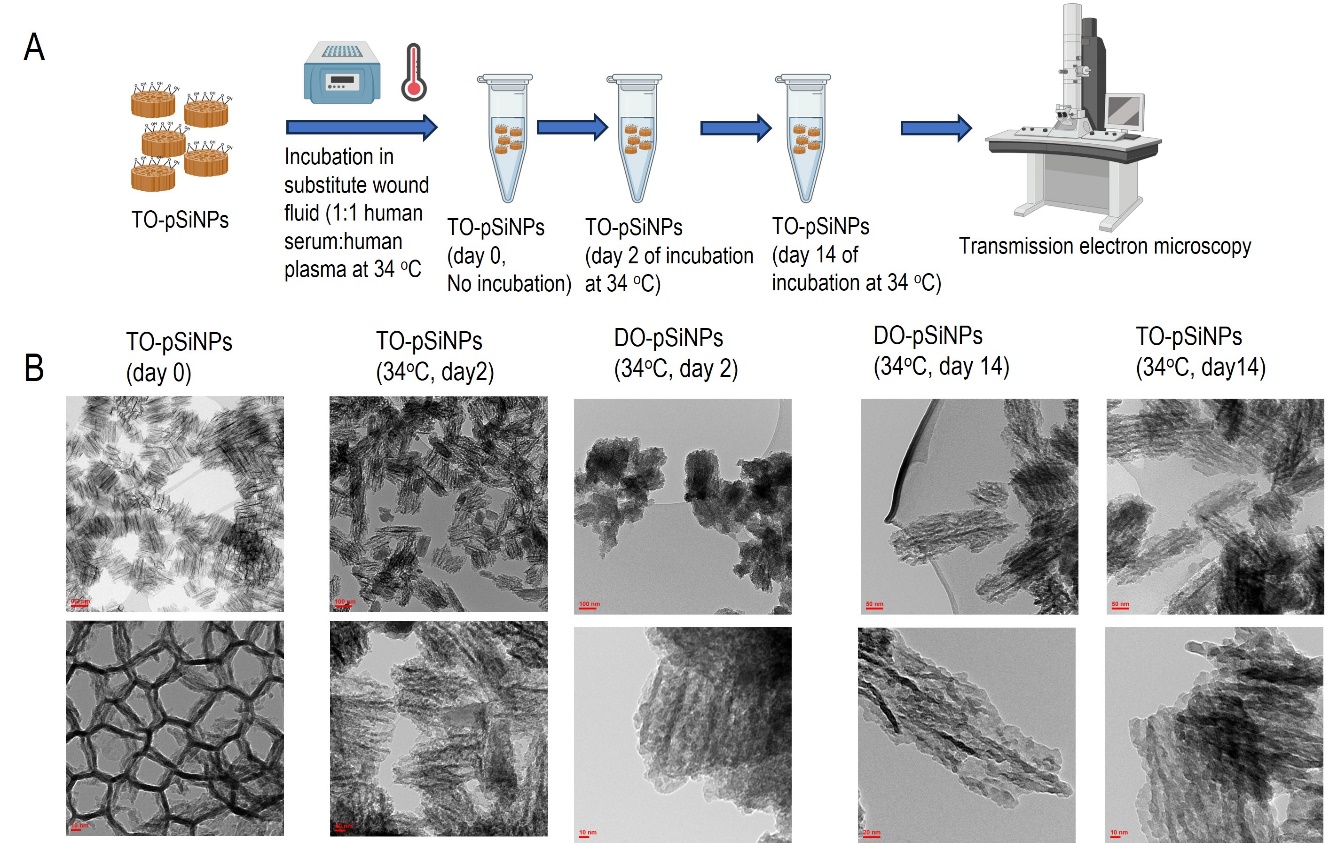


**Figure S2.** **Schematic illustration of porous silicon degradation test**. TO and DO pSi NPs were incubated in simulated wound fluid (SWF) for 14 days at 37 °C, then assessed by transmission electron microscopy for structural integrity.


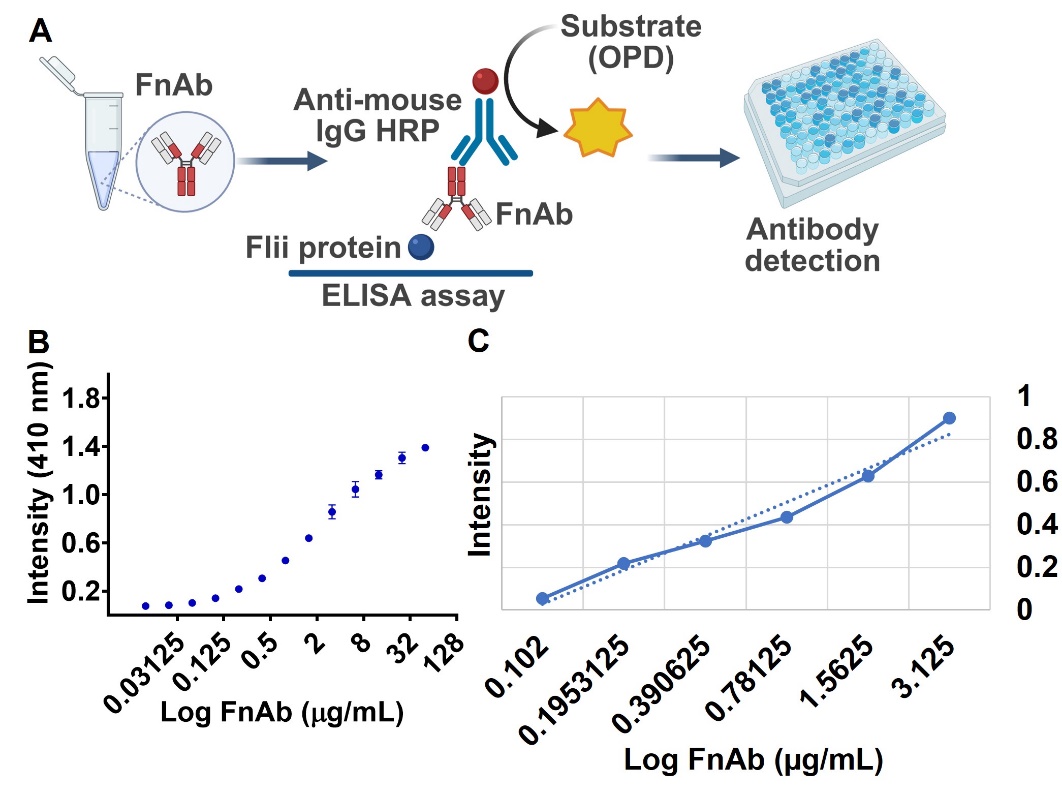


**Figure S3. Indirect ELISA assay detects functional FnAb released from pSi NPs**. (A) Illustration showing the assay which is based on the capacity of functional FnAb to bind to recombinant Flightless I protein (partial sequence). Reproduced with permission from BioRender. (B) FnAb was quantified using a calibration curve and measuring absorbance at 410 nm (n=3). The calibration curve was obtained for FnAb prepared in SWF (shown) or matching whichever buffer was used for FnAb release from pSi NPs. (C) The calibration curve linear range which was used to determine unknown FnAb release concentrations.

**Table S1.** Evaluation of mathematical models fitting for FnAb release kinetics from TO pSi NP and DO pSi NP

| Model | TO pSi NP R² | DO pSi NP R² | Other Parameters |
| --- | --- | --- | --- |
| Zero-order | 0.66 | 0.79 | k₀ = 9.15 (TO pSi NP), k₀ = 8.65 (DO pSi NP) |
| First-order | 0.97 | 0.94 | k₁ = 0.50 (TO pSi NP), k₁ = 0.29 (DO pSi NP) |
| Higuchi | 0.85 | 0.94 | k = 31.54 (TO pSi NP), k = 29.17 (DO pSi NP) |
| Korsmeyer-Peppas | 0.98 | 0.98 | n = 0.25 (TO pSi NP), n = 0.34 (DO pSi NP) |

**Table S2.** Mathematical models, their equations, mechanisms, and key parameters used for release kinetics analysis

| **Model name** | **Model equation** | **Description of mechanism** | **Parameters** |
| --- | --- | --- | --- |
| Zero-order | $m_{t}=m_{0}+k_{0}$ | Constant drug release/or particle degradation | m_0_​, m_t_, k_0_, t |
| First-order | $m_{t}=m_{0}e^{k_{1}t}$ | Concentration-driven diffusion (release rate directly proportional to payload concentration) | m_0_​, m_t_, k_0_, t |
| Higuchi | $m_{t}=kt^{\frac{1}{2}}$ | Drug dissolution and diffusion | m_0_​, m_t_, k, t |
| Korsmeyer-Peppas | ${m_{t}}/{m_{\infty}}=kt^{n}$ | unknown or multiple release mechanisms involved (including diffusion) | m_t_, m_∞_, k, t |


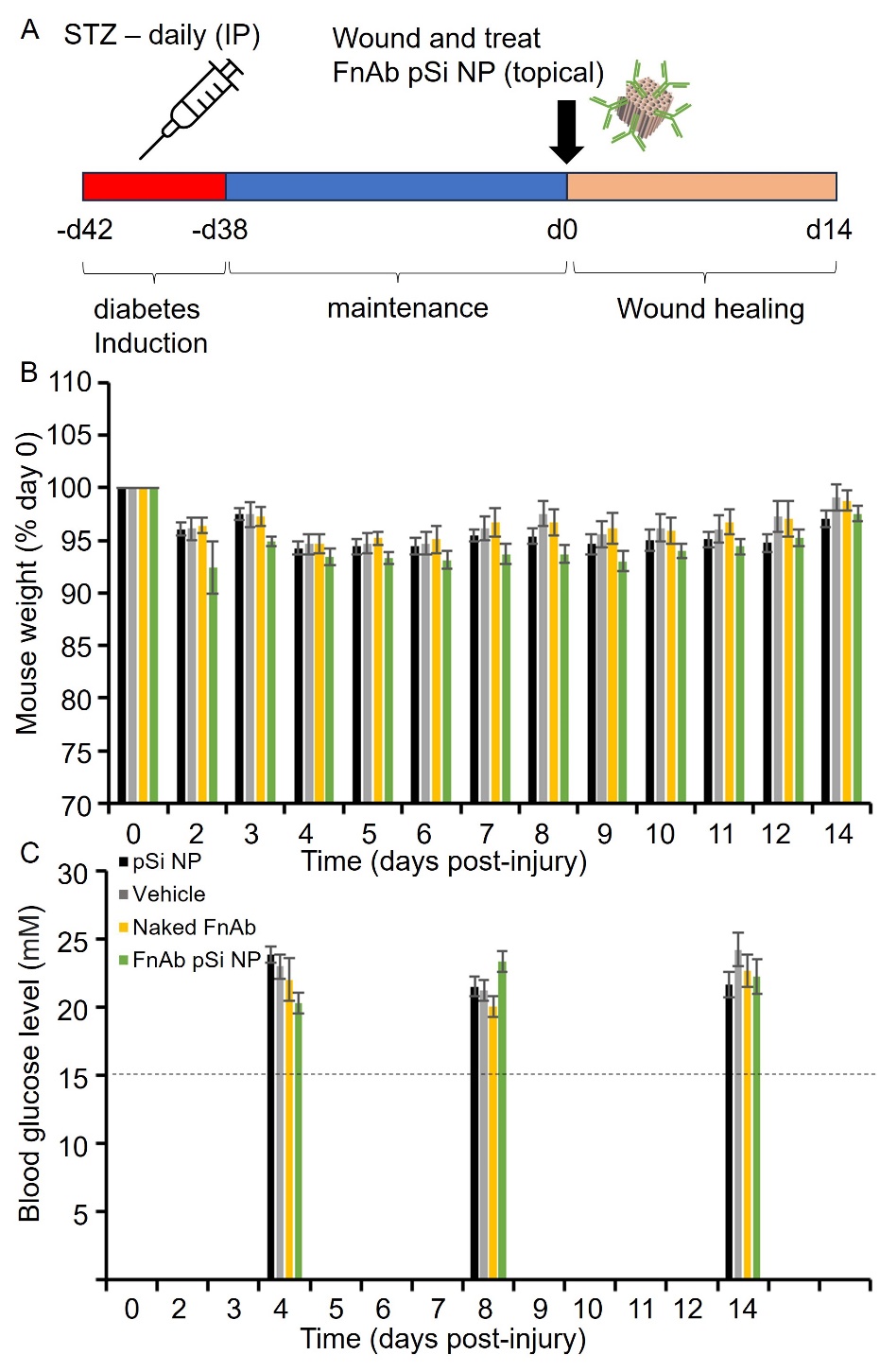


**Figure S4. Induction of type 1 diabetes in BALB/c mice**. (A) Schematic showing the protocol to induce diabetes with repeated low-dose STZ infusion and then excisional wound and treat with FnAb pSi NP. (B) Mouse weights recorded from day 0-14 post-injury. (C) Blood glucose levels were routinely measured from tail vein bleeds. Diabetes was defined as ≥ 15 mM (dashed line). Data presented in (B, C) as mean ± SEM (n=8 per group).


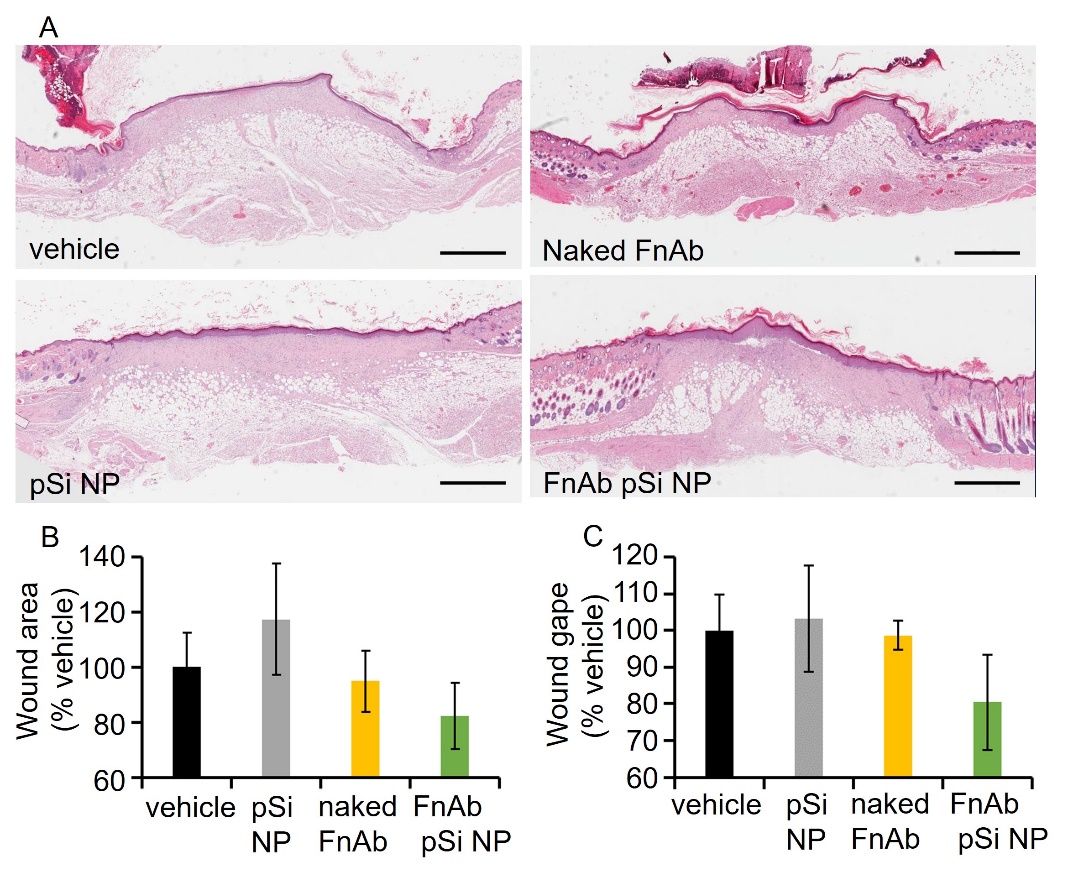


**Figure S5. FnAb pSi NPs trend towards improved wound gape and wound area in STZ diabetic mice**. Representative H&E stained tissue section images (A), quantification of wound area (B) and gape (C) of FnAb pSi NP-treated excisional wounds at day 14 post-injury. Data presented as a percentage of vehicle, mean ± SEM (n=8 per group). Size bars = 1 mm. All pairwise comparisons were analyzed by Student’s t-test (two-sided, nonpaired). Bonferroni correction was used when multiple pairwise tests were performed.


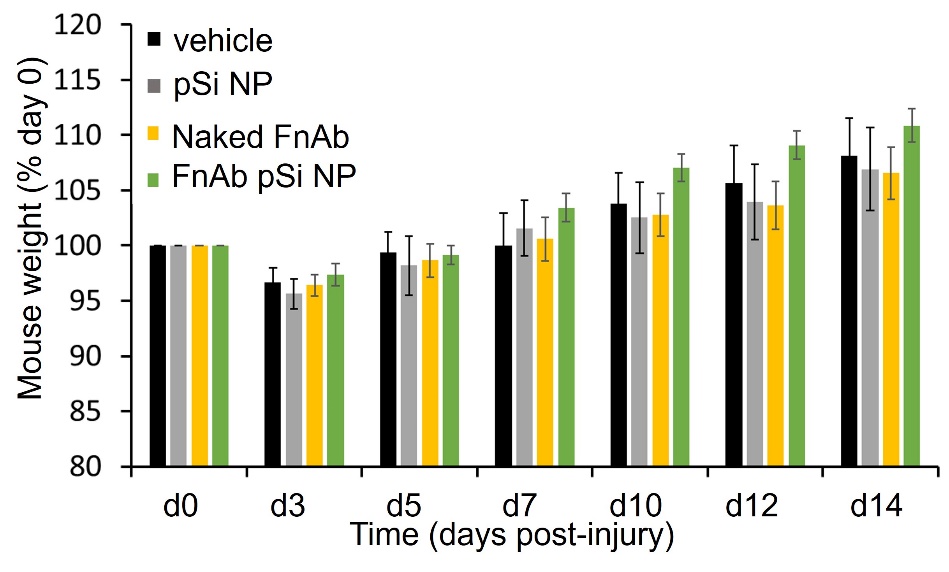


**Figure S6. Weights of type 2 diabetic mice post-injury**. Mouse weights recorded from day 0-14 post-injury. Data presented as mean ± SEM (n=8 per group).
